# Evidence-based clinical practice guidelines for IgA nephropathy 2014

**DOI:** 10.1007/s10157-015-1223-y

**Published:** 2016-04-20

**Authors:** Yukio Yuzawa, Ryohei Yamamoto, Kazuo Takahashi, Ritsuko Katafuchi, Makoto Tomita, Yoshihide Fujigaki, Hiroshi Kitamura, Masashi Goto, Takashi Yasuda, Mitsuhiro Sato, Maki Urushihara, Shuji Kondo, Shoji Kagami, Yoshinari Yasuda, Hiroyuki Komatsu, Miki Takahara, Yasuaki Harabuchi, Kenjiro Kimura, Seiichi Matsuo

**Affiliations:** Fujita Health University School of Medicine, Toyoake, Japan; Osaka University Graduate School of Medicine, Suita, Japan; National Fukuoka-Higashi Medical Center, Koga, Japan; Teikyo University School of Medicine, Tokyo, Japan; Chiba East Hospital, Chiba, Japan; Kyoto Medical Center, Kyoto, Japan; Kichijoji Asahi Hospital, Tokyo, Japan; JCHO Sendai Hospital, Sendai, Japan; Tokushima University Graduate School, Tokushima, Japan; Nagoya University Graduate School of Medicine, Nagoya, Japan; University of Miyazaki Hospital, Miyazaki, Japan; Asahikawa Medical University, Asahikawa, Japan; St. Marianna University School of Medicine, Kawasaki, Japan

## Preface

### 1. Background of this guideline

Immunoglobulin A (IgA) nephropathy (IgAN) is the most common primary glomerulonephritis, and patients typically require dialysis when the disease progresses to end-stage renal failure. As the incidence of IgAN is high in Asian populations, including Japanese, establishing a treatment strategy in Japan is strongly warranted. In 1995, the joint committee of the Special Study Group of the Progressive Renal Dysfunction Research Group of the Ministry of Health Labour and Welfare (MHLW) and the Japanese Society of Nephrology (JSN) developed the Clinical Practice Guides for IgAN for the first time. Its second version was published with a partial amendment in 2002. The third version, published in 2011, analyzed data from a multicenter study conducted mainly by the Research Group for IgAN in the Progressive Renal Dysfunction Research Group of MHLW to propose a novel prognostic classification (risk stratification for dialysis), adding clinical severity to histological severity. These clinical practice guides present clear prognostic criteria and treatment guidelines according to the criteria. Therefore, these guides have been widely used in clinical practice or pathological diagnosis, and they have contributed to the diagnosis and treatment of IgAN in Japan.

Meanwhile, Kidney Disease: Improving Global Outcomes (KDIGO) internationally published the Clinical Practice Guidelines for Glomerulonephritis in 2011. Recommendation grades based on the systematic review of clinical studies and the quality of evidence as a basis for determination of the strength of the recommendations are shown in the KDIGO Clinical Guidelines for Glomerulonephritis. IgAN is described in Chap. 10. However, careful evaluation was required to verify whether the KDIGO Clinical Guidelines for Glomerulonephritis was applicable to the actual clinical situation of IgAN in Japan, because in Japan, IgAN has been detected in routine checkups in the early stage, prognosis of IgAN has been classified in many cases according to the third version of the Clinical Guides for IgAN, and tonsillectomy has been performed in many cases. Therefore, establishing practice guidelines for IgAN that are adjusted to the situation in Japan is warranted. Responding to this need, the Progressive Renal Dysfunction Research Group of MHLW and JSN decided to develop the evidence-based Clinical Guidelines for IgA Nephropathy 2014. Thus, they established the Clinical Guidelines for IgA Nephropathy 2014 Advisory Committee. Against this background, the Clinical Guidelines for IgA Nephropathy 2014 was published. It is the first-ever-published comprehensive guideline only focusing on IgAN.

### 2. The intended purpose, anticipated users, and predicted social significance of the guidelines

The purpose of the Clinical Guidelines for IgA Nephropathy 2014 was to define evidence-based clinical guidelines that reflect the clinical situation of IgAN in Japan. This guideline is developed to provide answers to clinical questions (CQ) that nephrologists may encounter in the clinical practice for the treatment of IgAN. Each answer is shown as a statement, and recommendation grades based on the evidence-based levels are noted for each statement in the Treatment section. It was not aimed at creating an exhaustive textbook but at supporting clinical decisions by answering questions raised by nephrologists in clinical practice and establishing a standard treatment. With the aim of comprehensively supporting nephrologists in the treatment of IgAN in clinical settings, the Clinical Guidelines for IgA Nephropathy 2014 Advisory Committee independently evaluated the results of principal randomized parallel-group clinical trials published to date and presented the scheme of indications for preventive intervention of renal dysfunction progression in this guideline. Now, patients with IgAN at any stage can be treated by using this guideline in combination with the Evidence-based Practice Guideline for the Treatment of Chronic Kidney Disease (CKD). The Clinical Guidelines for IgA Nephropathy 2014 also describe the characteristics and treatment of pediatric IgAN.

Evidence from the literature can provide information but is not a substitute for the specialized skills and experiences of individual physicians. Whether a particular statement applies and how it applies to a particular patient depends on the specialist abilities of each physician. The times demand that medical care shift from a one-size-fits-all approach to a tailor-made approach. Clinical guidelines are not supposed to impose a uniform style of care on physicians. Each physician needs to determine what kind of care each patient needs based on an understanding of the content of clinical guidelines. As such, these guidelines are not intended to limit physicians to certain forms of medical behavior but were created to assist them in exercising their discretion to decide the type of care to be provided. In addition, it should be stated clearly that these guidelines are not criteria for deciding physician–patient conflicts or medical malpractice lawsuits.

### 3. Patients within the scope of the guidelines

These guidelines apply to IgAN patients of all ages. In the KDIGO Clinical Practice Guidelines, atypical IgAN is a minimal-change condition, in which IgA deposits are observed in the mesangium, is an acute renal insufficiency with gross hematuria, and presents as crescentic IgAN. Accordingly, the present guidelines recommend the aforementioned conditions should be treated as special types of IgAN. A summary of pediatric diagnostic and treatment modalities was also included.

Cases requiring CKD management were addressed based on the “2013 CKD Clinical Guideline Based on Evidence.” Finally, pregnancy-related items were, as a rule, not included.

### 4. Preparation procedure

Creating evidence-based guidelines first requires the enormous task of gathering and evaluating evidence. We would like to sincerely thank the members of the IgAN Clinical Guidelines Working Group for their dedication and effort. (show list of contributors)

The first meeting of the clinical guidelines working group was held on September 23, 2011. The group was led by Dr. Kenjiro Kimura of the St. Marianna University School of Medicine, who explained the significance of creating the guidelines and the procedures for the task. The IgAN clinical guidelines committee first met on October 14, during which members of the IgAN Clinical Guidelines Working Group set about creating the guidelines based on a shared understanding. In effect, this was the initial stage of the drafting of the guidelines. The MINDS handbook for creating clinical guidelines was followed, and the Delphi method was used in composing CQ, which is the core of the guidelines. Recommendation grades were determined by an informal consensus. As a rule, PubMed records up to July 2012 were used to search the literature. If necessary, important studies from after this date were included, with reasons given.

The IgAN clinical guidelines committee met 12 times, although the group also often communicated through e-mail. Through this process, the initial CQ and text items were revised as needed, and a few deletions and additions were made. From September 13 to October 13, 2013, each part was reviewed by two designated referees and two designated academic societies. Simultaneously, public comments were solicited from members of the Japanese Society of Nephrology (JSN). The manuscript was then revised based on the referees’ opinions and public comments. The IgAN clinical guidelines committee met on January 26, 2014, to examine the revised manuscript. Afterward, additional revisions were made as needed until a final draft was obtained. The guidelines, as well as responses to the referees’ opinions and public comments, were posted on the JSN Web site.

### 5. Contents of the guideline

The guidelines are composed of three chapters as follows: I. Concepts, II. Diagnosis, and III. Epidemiology and Prognosis. These guidelines were created in tandem with the “2013 CKD Clinical Guideline Based on Evidence,” and so were written by the same authors.

Items in the structured abstracts attached to the guidelines were standardized to contain the reference number, reference title, Japanese title, evidence level, author names, journal name, publication year/page, objectives, study design, subject patients, intervention factors, primary outcomes, results, and discussion.

### 6. Evidence levels and recommendation grades

Evidence levels were evaluated in a manner similar to that described in the “2013 CKD Clinical Guideline Based on Evidence.”

- [Evidence levels]
- Level 1: Systematic review/meta-analysis.
- Level 2: At least 1 randomized controlled trial (RCT).
- Level 3: A non-RCT.
- Level 4: An analytical epidemiologic study (cohort study or case–control study) or a single-arm intervention study (no controls).
- Level 5: A descriptive study (case report or case series).
- Level 6: Opinion of an expert committee or an individual expert, which is not based on patient data.

Evidence levels for meta-analyses and systematic reviews were determined from the designs of the studies on which they were based. If the underlying studies had mixed designs, consensus was reached to adhere to the lowest level (e.g., a meta-analysis of cohort studies would be level 4, as would a meta-analysis that included both RCT and cohort studies).

Consensus was also reached to assign evidence level 4 to all RCT subanalyses and post hoc analyses. Therefore, an RCT with a clear primary outcome would be considered level 2, while a subanalysis or post hoc analysis of this RCT would be considered level 4.

The following recommendation grades were assigned to statements about treatments, which were based on the level of evidence for each statement.

- [Recommendation Grades]
- Grade A: Strongly recommended because the scientific basis is strong.
- Grade B: Recommended because there is some scientific basis.
- Grade C1: Recommended despite having only a weak scientific basis.
- Grade C2: Not recommended because there is only a weak scientific basis.
- Grade D: Not recommended because scientific evidence shows treatment to be ineffective or harmful.

As a rule, standard treatments in Japan were recommended, but eligibility for health insurance coverage was not necessarily required. Drugs ineligible for insurance coverage were denoted as such. Recommendation grades were assigned to statements about treatment-related CQ. In addition, questions such as “To which subgroup would this be recommended?” and “To which subgroup would this not be recommended?” were addressed whenever possible. Recommendation grades were decided through consultations among the working group members by considering the tradeoffs between and balance of benefits, damage, side effects, and risk. If differing views existed among the referees or in the public comments, the group reexamined the area through an exchange of opinions. The reasons for choosing a recommendation grade and the decision-making process involved were described in the commentary, as a rule.

### 7. Issues on the preparation of this guideline

Although evidence concerning IgAN is gradually increasing in Japan, it is still insufficient, which means that these guidelines were heavily influenced by evidence from Europe and the United States. Whether the results of clinical research from the West can be applied as is to Japan is a question that deserves careful consideration. Only a few large clinical studies have been performed on IgAN even in the West, so the quality of evidence is limited. In creating these guidelines, we strove to ensure that they would not deviate greatly from the clinical practice in Japan.

### 8. Financial sources and conflict of interest

The funds used in creating the guidelines were provided by a research group on progressive kidney disorders funded by the Ministry of Health, Labour, and Welfare’s research project for overcoming intractable diseases. These funds were used to pay for transportation to and from meetings, to rent space for meetings, and for box lunches and snacks. The committee members received no compensation. Everyone involved in creating the guidelines (including referees) submitted conflict-of-interest statements based on academic society rules, which are managed by JSN. Opinions were sought from multiple referees and related academic societies to prevent the guidelines from being influenced by any conflicts of interest. Drafts were shown to the society members, and revisions were made based on their opinions (public comments).

### 9. Publication and future revisions

The guidelines are to be published in Japanese-language journal of JNS and concurrently released in book form by Tokyo Igakusha. They will also be posted on the JSN Web site and on the MINDS Web site of the Japan Council for Quality Health Care.

## I. Introduction

### 1. Definition and background

IgA nephropathy (IgAN, also known as Berger’s disease) is a disease characterized by urinary findings suggesting glomerulonephritis; predominantly, IgA is deposited in the glomeruli, with no evidence of other underlying disease. Glomerular hematuria and proteinuria are urinary findings that suggest glomerulonephritis. Renal biopsy findings, which are required for confirming the diagnosis of glomerulonephritis, include IgA deposits mainly in the glomerular mesangium and occasionally in the capillary loops. In many cases, C3 is also co-deposited. The rate of progression to end-stage renal disease (ESRD) is approximately 40 % at 20 years after diagnosis. Treatment may include therapy with renin-angiotensin system (RAS) blockers, antiplatelet agents, oral corticosteroids, fish oil, or non-steroidal immunosuppressive agents; steroid pulse therapy; or tonsillectomy. The therapeutic effects of each have been examined, but an effective treatment regimen is yet to be established.

### 2. Pathogenesis and pathophysiology

#### 1. Overview

In patients with IgAN, for some unknown reason, the level of nephritogenic IgA1 increases in the circulation and is deposited in the mesangium, leading to glomerular damage. The exact mechanism of IgAN is unknown. Exacerbation in patients with upper respiratory infections has been well known, thereby suggesting changes in mucosal immunity are involved in the pathogenesis. Many other mechanisms are also involved in the pathogenesis of IgAN: production and increase of pathogenic IgA1, IgA1 deposition into the glomeruli, proliferation of mesangial cells and matrix expansion from the deposits, and persistent and progressive glomerulonephritis. A genetic predisposition may also play a role in the pathogenesis of IgAN.

#### 2. Genetics

Most cases of IgAN are sporadic, but approximately 10 % are familial cases. Regional and ethnic differences are also seen in sporadic IgAN, and polygenic inheritance is found to be involved. The responsible genes differ between sporadic and familial IgAN, and genetic involvement in the disease may be monogenic or polygenic, depending on the individual or family. Pedigrees with autosomal dominant transmission have also been reported. Recent genome-wide association studies (GWAS), in which whole-gene association analysis is applied, have highlighted important findings.

#### 3. Abnormal IgA molecules

Approximately half of all patients with IgAN have elevated levels of serum IgA, associated with increased IgA1 production from the bone marrow and/or mucosa. The IgA1 deposited in the glomeruli is from the circulating IgA1, and the serum IgA1 in patients with IgAN has been analyzed in detail. Serum IgA1 has clustered O-linked glycans on its hinge-region. Aberrantly glycosylated IgA1, i.e., galactose (Gal)-deficiency in some O-glycans, is increased in serum IgA1 and IgA1 extracted from the glomeruli.

#### 4. Mucosal immunity

Some patients with IgAN have worsening clinical symptoms with macroscopic hematuria after upper respiratory or gastrointestinal infections, thereby suggesting a relationship between IgAN and mucosal immunity. Increased polymeric IgA1 in the circulation of patients with IgAN after an upper respiratory infection, and improvement in nephropathy after tonsillectomy, have been reported. Abnormal mucosal reactions may increase circulating polymeric IgA1, thus leading to glomerular deposition.

#### 5. IgA1 glomerular deposition

IgA1 is selectively deposited in patients with IgAN; IgA1 has an affinity to the mesangium, especially the dimeric and polymeric IgA1 with J chains, and acidic IgAN containing λ light chains. In addition, the deposited IgA1 has abnormal hinge-region O-linked glycans. High-molecular-weight IgA1, including serum polymeric IgA1, is deposited in the glomeruli.

#### 6. Glomerular damage

Mesangial cell activation and complement activation through IgA deposition lead to glomerulonephritis, followed by podocyte and renal tubular injury. Humoral factors released from the mesangial cells play an important role in podocyte injury and tubulointerstitial damage (glomerulus-podocyte-renal tubule cross-talk).

## II. Diagnosis

### 1. Diagnosis

Although various attempts have been made to diagnose IgAN according to clinical findings, IgAN is diagnosed on the basis of renal biopsy findings. On immunohistochemical study, IgAN is defined as dominant staining with IgA in a glomerulus. Histological findings such as in Henoch-Schönlein purpura nephritis (IgA vasculitis), lupus nephritis, and nephritis associated with liver cirrhosis and rheumatoid arthritis are similar to those in IgAN; therefore, a differential diagnosis should be based on clinical characteristic and laboratory data.

### 2. Clinical manifestations and laboratory findings

#### 1. Clinical symptoms and physical examination findings

Most cases of IgAN are characterized by asymptomatic urinary abnormalities. Acute nephritic syndrome or evaluation of edema due to nephrotic syndrome may also lead to the diagnosis of IgAN. Macroscopic hematuria occurs in conjunction with an acute upper respiratory infection in some cases. However, macroscopically, no specific findings related to IgAN are observed in the palatine tonsils. In IgAN patients with progressively deteriorating renal function, moderate to severe proteinuria, followed by hypertension and a decline in renal function usually occurs in order.

#### 2. Urinalysis findings

Most patients with IgAN have asymptomatic hematuria or proteinuria; this urinary abnormality leads renal biopsy. Therefore, urinalysis is essential for the diagnosis of IgAN. Currently, routine urinalysis will not show findings specific for IgAN. The Clinical Guidelines for IgA Nephropathy (ver. 3) state that persistent microscopic hematuria is an essential finding, and intermittent or persistent proteinuria is a frequently associated finding. Moreover, macroscopic hematuria may be an incidental finding. To confirm reproducibility and persistence of the urinary abnormality, the results of at least 3 urinalyses should be considered before confirming the diagnosis. Furthermore, urinalysis on at least 2 of these occasions should, besides qualitative dipstick testing, also include analysis of urinary sediment. No urinary biomarker has yet been established to diagnose IgAN.

#### 3. Blood biochemistry findings

No specific blood test results have been established for a diagnosis of IgAN. A frequent finding in about half of patients is elevated serum IgA levels (≥315 mg/dL). In addition, a high serum IgA/C3 ratio is also reported as a useful finding for differential diagnosis. At the research level, serum levels of aberrantly glycosylated IgA1, related immune complexes, and corresponding antibodies are reported to be useful as blood biomarkers of IgAN.

#### 4. Indications for renal biopsy

Clinically, persistent microscopic hematuria and proteinuria, elevated serum IgA level, a high serum IgA/C3 ratio, and macroscopic hematuria with upper respiratory infection are strong indicators of IgAN. However, a renal biopsy is essential for a definitive diagnosis of IgAN. In addition, a renal biopsy for histopathological examination is also important for patient management, because clinical and laboratory findings alone are insufficient for assessing prognosis and selecting the appropriate treatment modality. In patients who only have asymptomatic microscopic hematuria or trace proteinuria, patient management strategy will rarely be altered by histological findings, so a renal biopsy may be optional. However, renal biopsy should be considered to differentiate between thin basement membrane disease and Alport syndrome.

#### 5. Features of childhood IgA nephropathy

Childhood IgA nephropathy in Japan is usually found on urinary screening in schools, often leading to prompt diagnosis and initiation of treatment.

### 3. Pathological findings

IgAN is defined as glomerulonephritis with predominant IgA deposits in the mesangium, and kidney biopsy is essential for its diagnosis. Histological changes in IgAN mainly involve the mesangium, but various glomerular lesions other than those in the mesangium also occur; for instance, tubular, interstitial, and vascular lesions may also develop. Precise definitions have recently been proposed for the various lesions that develop in IgAN, and examination of the lesions based on these definitions is now recommended. Pathologic diagnosis is important not only for diagnosing IgAN but also for assessing the prognosis of kidney function.

### 4. Classification

Classification should be useful for predicting prognosis and selecting an appropriate treatment regimen. Although various classifications have been reported so far, not one has achieved worldwide consensus. Recently, the Clinical Guidelines for IgA Nephropathy (ver. 3) published in Japan 31) and histologic classification based on a multicenter case–control study on IgAN in Japan have been put forth (Table 1A–C). At the international level, the Oxford Classification has been published ( Table 2). Thus, the management of IgAN will be based on these guidelines and classification. Both classifications should be modified on the basis of the findings of further validation studies in the future.

**Table 1:** Histologic classification presented by a multicenter case–control study on patients with IgAN in Japan

Histological grade	% glomeruli with pathological variables** predicting progression to ESRD	Acute lesion only	Acute and chronic lesion	Chronic lesion only
A. Hitological grade				
H-Grade I	0–24.9 %	A	A/C	C
H-Grade II	25–49.9 %	A	A/C	C
H-Grade III	50–74.9 %	A	A/C	C
H-Grade IV	>75 %	A	A/C	C

Clinical grade	Proteinuria (g/day)	EGFR (ml/min/1.73 m^2^)		
B. Clinical grade				
C-Grade I	<0.5	–		
C-Grade II	0.5≤	60≤		
C-Grade III		<60		

Clinical grade	Histological grade			
	H-Grade I	H-Grade II	H-Grade III + IV	
C. Grading system for predicting progression to ESRD				
C-Grade I	Low	Moderate	High	
C-Grade II	Moderate	Moderate	High	
C-Grade III	High	High	Super high	

**Acute lesion (A): cellular crescent, tuft necrosis, fibrocellular crescent

Chronic lesion (C): global sclerosis, segmental sclerosis, fibrous crescent

Low risk group: *1 of 72 (1.4 %) of IgAN patients developed to ESRD in 18.6 years after RBx

Moderate risk group: *13 of 115 (11.3 %) of IgAN patients developed to ESRD in 11.5 (3.7-19.3) years after RBx

High risk group: *12 of 49 (24.5 %) of IgAN patients developed to ESRD in 8.9 (2.8–19.6) years after RBx

Super high risk group: *22 of 34 (64.7 %) of IgAN patients developed to ESRD in 5.1 (0.7–13.1) years after RBx

* The data from retrospective multicenter case–control study on IgAN (*n* = 287)

**Table 2:** Definitions of pathological variables used in the Oxford classification

Variable	Definition		Score
Mesangial hypercellularity	<4	Mesangial cells/mesangial area = 0	M0 ≤ 0.5
	4–5	Mesangial cells/mesangial area = 1	M1 > 0.5^a^
	6–7	Mesangial cells/mesangial area = 2	
	≧8	Mesangial cells/mesangial area = 3	
		The mesangial hypercellularity score is the mean score for all glomeruli	
Segmental glomerulosclerosis	Any amount of the tuft involved in sclerosis, but not involving the whole tuft or the presence of an adhesion		S0—absent
			S1—present
Endocapillary hypercellularity	Hypercellularity due to increased number of cells within glomerular capillary lumina causing narrowing of the lumina		E0—absent
			E1—present
Tubular atrophy/interstitial fibrosis	Percentage of cortical area involved by the tubular atrophy or interstitial fibrosis, whichever is greater		T0—0–25 %
			T1—26–50 %
			T2—>50 %

^a^Mesangial score should be assessed in periodic acid-Schiff-stained sections. If more than half the glomeruli have more than three cells in a mesangial area, this is categorized as M1. Therefore, a formal mesangial cell count is not always necessary to derive the mesangial score

### 5. Atypical forms of IgA nephropathy

#### 1. Minimal change nephrotic disease (MCD) with mesangial IgA deposits

Rarely, in some patients with nephrotic syndrome, the kidney biopsy shows minimal glomerular changes on light microscopy, and predominant glomerular deposits of IgA on immunohistochemical study. Because prompt, complete remission after corticosteroid therapy and the following clinical course, with frequent nephrotic syndrome relapses, are very suggestive of minimal change nephrotic disease (MCD), a coincidence of MCD and IgAN has been proposed as the most likely explanation for such cases. Nephrotic syndrome occurs in 5–25 % of all patients with IgAN, and the coincidence of MCD among these patients is 25–47 % (1.8–6 % of all patients with IgAN).

#### 2. Acute kidney injury (AKI) associated with macroscopic hematuria

Episodic macroscopic hematuria coinciding with mucosal infection is a hallmark of IgAN. The macroscopic hematuria usually resolves spontaneously in a few days, and kidney function usually recovers completely after the disappearance of macroscopic hematuria. However, in rare cases, the macroscopic hematuria is prolonged and acute kidney injury (AKI) develops. AKI occurs in less than 5 % of the patients with IgAN. Histologically, crescent formation and obstruction of tubules by red blood cell casts and tubular epithelial cell injury are frequently observed. AKI cannot be explained by the percentage of crescent formation in the glomerulus alone, and many studies have reported that AKI is mainly caused by red blood cell casts and the resulting renal tubular epithelial injury. In a majority of patients, kidney function returns to baseline after the disappearance of macroscopic hematuria, but incomplete recovery of kidney function has been reported in up to 25 % of the affected patients in long-term follow-up studies. Macroscopic hematuria lasting longer than 10 days is the most significant risk factor of persistent kidney impairment.

#### 3. Crescentic IgA nephropathy

Crescentic IgAN is defined by different studies according to the percentage of the glomeruli with crescent formation ranging between 10 and 80 % of the glomeruli with crescent formation. Crescentic IgAN was found to account for 5 % of all IgAN cases in a study that used a definition of crescentic IgAN as more than 30 % of the glomeruli with crescent formation and for 1.14 % of all IgAN cases in a study that used a definition of crescentic IgAN as more than 50 % of the glomeruli with crescent formation. The histopathological analysis yields not only active lesions such as widespread cellular crescents, endocapillary hypercellularity, and tuft necrosis, but also a varying degree of chronic lesions such as glomerular sclerosis and interstitial fibrosis. Clinical manifestations include rapidly progressive glomerulonephritis, hypertension, severe proteinuria, and frequently macroscopic hematuria. Steroid and cyclophosphamide therapy may be effective in crescentic IgAN, but their effectiveness remains controversial.

## III. Epidemiology, prognosis, and follow-up

### 1. Incidence and prevalence

In Japan, about one-third of all patients who undergo renal biopsy are diagnosed with IgAN. The incidence of IgAN is estimated to be 3.9 to 4.5 per 100,000 persons per year. An estimated 33,000 persons have IgAN (95 % CI 28,000–37,000).

### 2. Natural course

The 10-year renal survival rate in adult-onset IgAN is approximately 80–85 %. The 10-year renal survival rate in childhood-onset IgAN is over 90 %.

### 3. Changes in prognosis with changes in treatment guidelines

Various studies show that the prognosis of IgAN is better in patients diagnosed in the 1990s and later than in patients diagnosed before then, suggesting that changes in treatment guidelines for IgAN have been successful.

### 4. Clinical predictors of progression at the time of initial examination or renal biopsy

Clinical predictors of progression in patients with IgAN at the time of the initial examination or renal biopsy include amount of proteinuria, blood pressure levels, degree of renal dysfunction, and histological severity. Therefore, models to predict the renal prognosis from the time of the initial examination or renal biopsy have been developed with combinations of these factors and are used in prognostic predictions for IgAN. 35–39) However, along with the prolonged disease duration and progression of disease, the amount of proteinuria, blood pressure level, renal function, and histological lesions progressively deteriorate. Therefore, these factors may simply reflect the stage (grade) of disease. Factors indicating the progression rate of disease at each stage (grade) of IgAN have not been identified.

### 5. Clinical predictors of progression during follow-up

Because multiple renal biopsies are not feasible, the clinical predictors of progression of IgAN during follow-up are proteinuria, blood pressure, and hematuria. Both the mean proteinuria level and the mean blood pressure levels during follow-up have known to be stronger risk factors for ESRD than factors like amount of proteinuria, blood pressure level, degree of renal dysfunction, and histological severity of IgAN at the time of initial examination or renal biopsy. In particular, maintaining proteinuria at <1.0 g/day and blood pressure at <130/80 mmHg during follow-up are associated with improved renal prognosis.

### 6. Remission of urinary findings and its significance

In patients with IgAN, normalization of urinary findings during the natural course or after treatment, in other words, a remission of urinary findings defined as an improvement or disappearance of hematuria and proteinuria is reported to be associated with improved renal prognosis. However, remission of urinary findings has been variously defined to date. Therefore, the significance of remission of urinary findings during the natural course or after treatment in the renal prognosis in patients with IgAN is unclear. Studies have been initiated to standardize the definition of remission, evaluate the therapeutic effectiveness of treatment regimens based on a standard definition of remission, and clarify the significance of the remission of urinary findings. Furthermore, patients who have achieved remission of urinary findings may later again experience worsening of urinary findings, in other words, a recurrence. Recurrence after remission of urinary findings has also not been defined, and its significance is also unclear.

### 7. Follow-up

At present, there is no robust evidence for the follow-up protocols of IgAN to improve renal prognosis. Currently, both the degree of renal dysfunction and amount of proteinuria are used as markers in follow-up protocols. As renal dysfunction worsens and proteinuria level increases, careful monitoring of the clinical course and treatment effectiveness at shorter follow-up intervals is recommended. In addition, follow-up intervals should be adjusted according to renal biopsy findings, urinary findings, achieved blood pressure levels, the rate of progression of renal dysfunction, and the type of treatment regimen. Moreover, urinary findings may improve over a period of years after various treatments, while recurrence after improved urinary findings may occur after long period of time. Therefore, long-term follow-up is strongly recommended in patients with IgAN, even in patients with only mild urinary abnormalities.

## IV. Treatment

### 1. A summary of management of IgAN in adults, with a focus on prevention of renal dysfunction

**Fig. 1:**
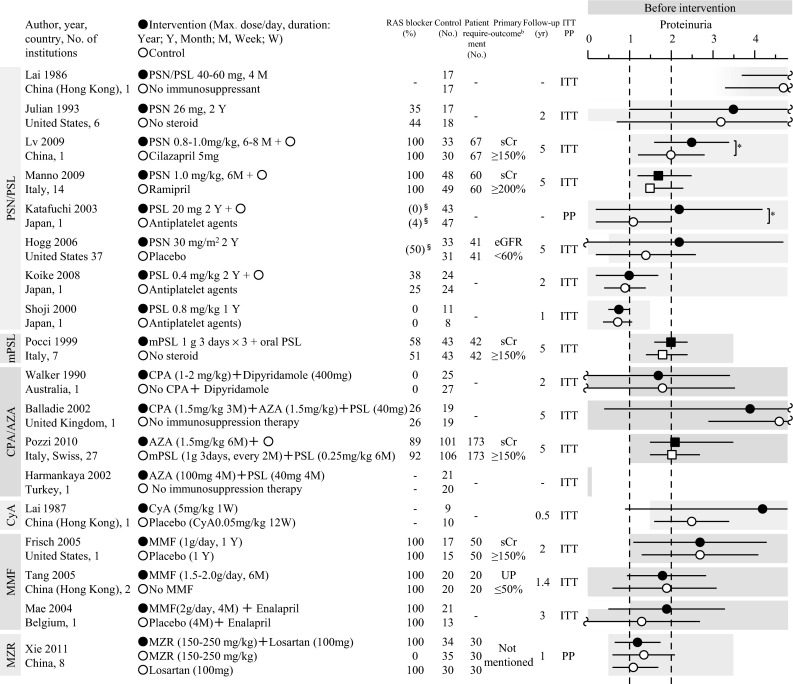

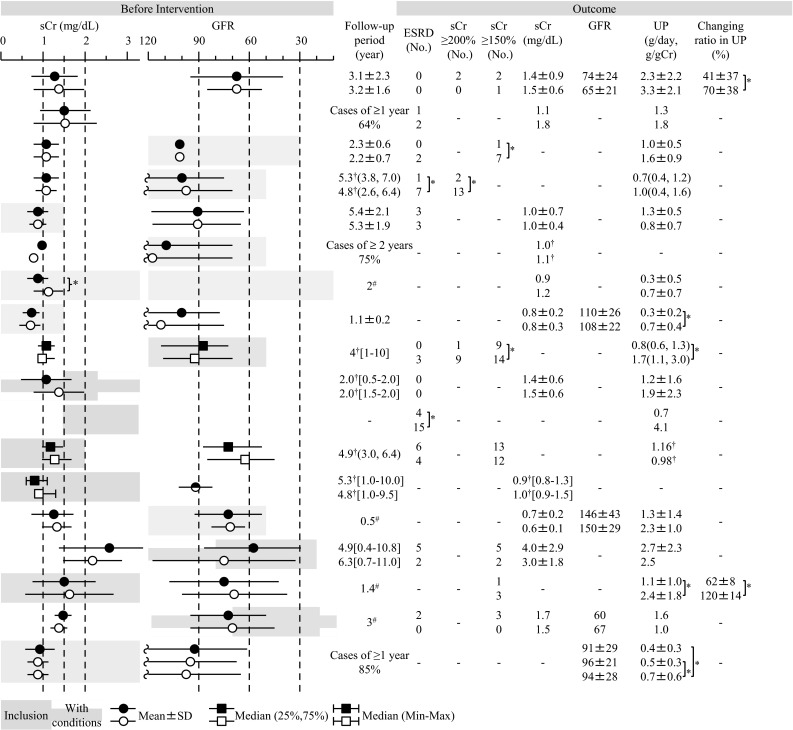
The summary of randomized controlled trials of corticosteroids and immunosuppressive agents in adult patients with IgAN

**Fig. 2:**
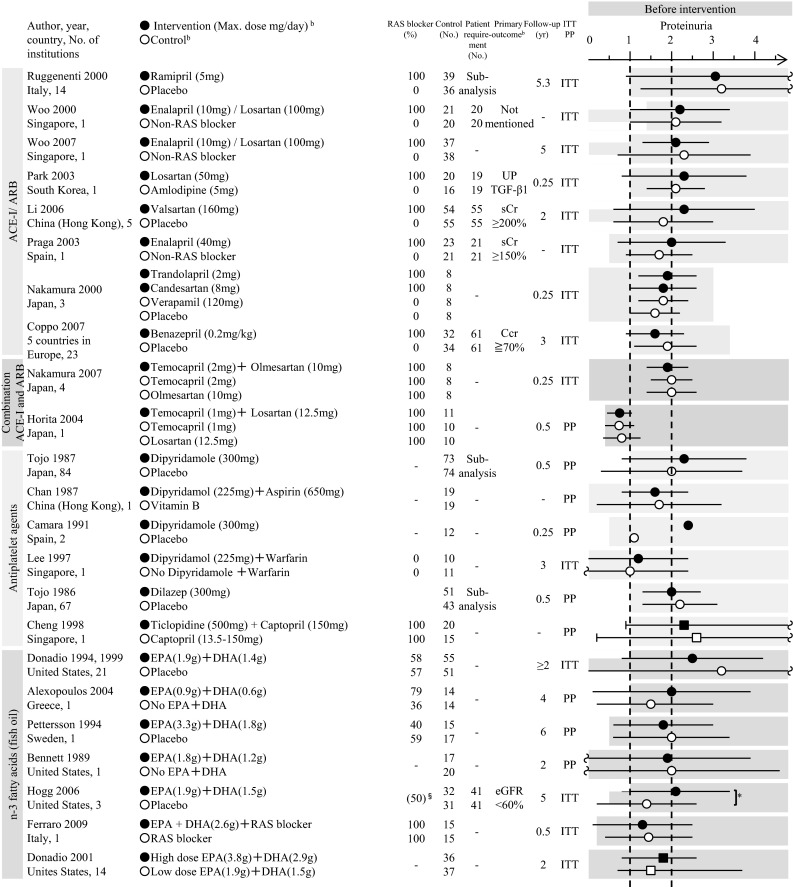

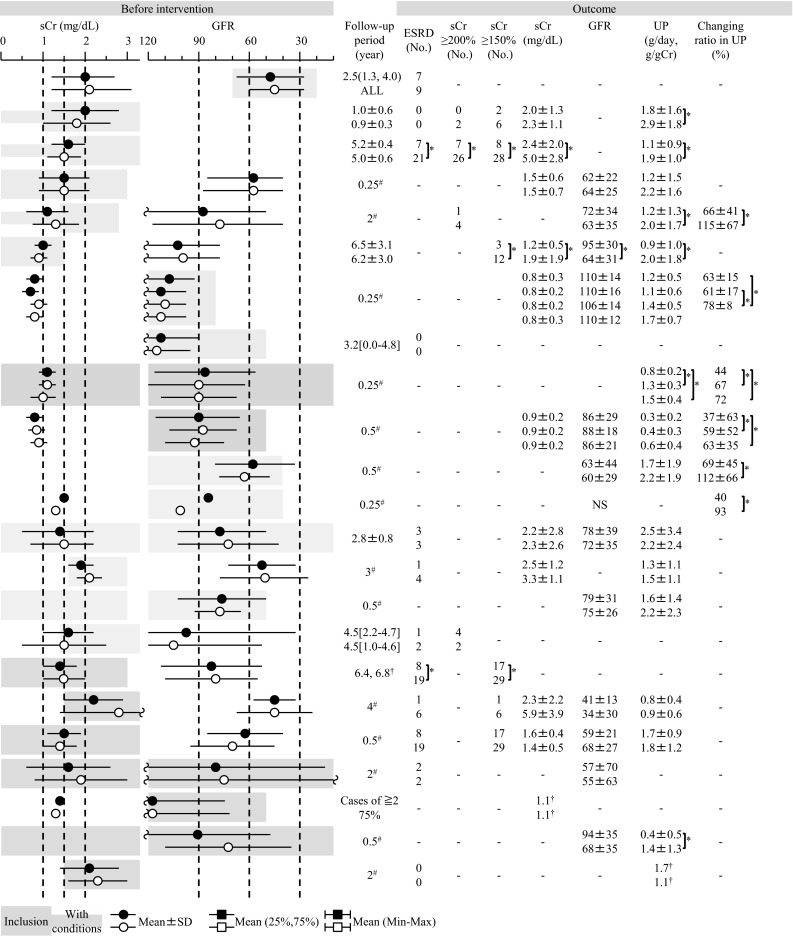
The summary of randomized controlled trials of RAS blockers, antiplatelet agents, and fish oils in adult patients with IgAN

**Fig. 3:**
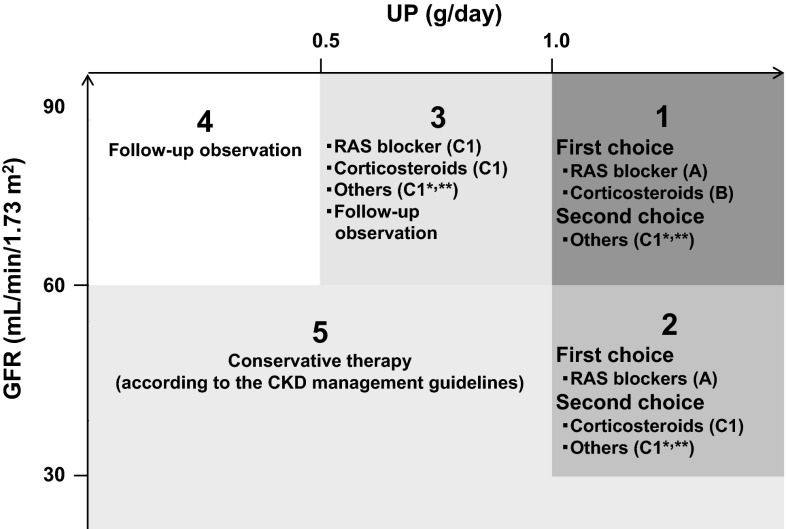
An outline of treatment of IgAN in adults with a focus on prevention of renal dysfunction (based on randomized controlled trials for IgAN) This figure shows the indications for treatment intervention, based mainly on the results (Figs. 1, 2) of RCTs, often focusing on renal function and amount of urinary protein excreted as patient inclusion/exclusion criteria. In actual clinical practice, besides renal function and urinary protein level, other factors such as renal histopathological findings and age should also be considered to carefully decide the indications for these treatment interventions. Others *: Tonsillectomy (combined with high-dose pulse corticosteroid therapy) and therapy with non-steroidal immunosuppressive agents, antiplatelet agents, and n-3 fatty acids (fish oil). CKD management guidelines **: The Japanese Society of Nephrology Evidence based Clinical Practice Guideline for CKD 2013: Hypertension (Chap. 4), salt intake (Chaps. 3, 4), lipid disorders (Chap. 14), glucose intolerance (Chap. 9), obesity (Chap. 15), smoking (Chap. 2), anemia (Chap. 7), CKD mineral and bone disorders (CKD-MBD, Chap. 8), and metabolic acidosis (Chap. 3) should also be managed as necessary

In Japan, the major potential treatment modalities for adult IgAN are the use of RAS blockers, corticosteroids, non-steroidal immunosuppressive agents, antiplatelet agents, and n-3 fatty acids (fish oil) and tonsillectomy (with corticosteroid pulse therapy). We evaluated the reduction of proteinuria and preservation of kidney function caused by therapeutic interventions based on the results of several randomized controlled trials (RCTs), as shown in Figs. 1 and 2. Consequently, the following guidelines have been developed for the treatment of patients with IgAN: To suppress IgAN progression, treatments should be based on renal function, urinary protein, age, and renal histopathological findings. Interventions to optimize blood pressure, salt intake, lipid and glucose metabolism, body weight, and smoking habits should be considered, if necessary (Fig. 3).

### 2. Clinical questions (CQs) about immunosuppressive therapy (adults)



To evaluate the effectiveness of corticosteroid therapy for treating IgAN, patients with IgAN with urinary protein level ≥1 g/day and CKD stage G1-2 were enrolled in a randomized controlled trial (RCT). A short course of high-dose oral steroid therapy (prednisolone at a dose of 0.8–1.0 mg/kg for about 2 months, followed by gradual tapering over about 6 months), along with concomitant administration of RAS blockers, improved the renal function, as seen in 2 different studies, so this regimen is recommended. Steroid pulse therapy (methylprednisolone 1 g for 3 days, every other month, 3 times + prednisolone 0.5 mg/kg, every other day, for 6 months) also improved the renal function; however, this improvement was reported in 1 study only. The validity of this result needs to be reconfirmed. Therefore, high-dose oral steroid therapy may be effective in reducing urinary protein levels for patients with IgAN with urinary protein level of 0.5–1.0 g/day and CKD stage G1-2. Nevertheless, further investigation is necessary.

In a retrospective cohort study, Hotta et al. reported that tonsillectomy combined with steroid pulse therapy normalized urinary findings, which are predictive factors for renal failure. Additionally, in a non-randomized comparative study, Komatsu et al. reported that the normalization rate of urinary findings was higher with tonsillectomy combined with steroid pulse therapy than with steroid pulse therapy alone. However, the level of evidence is regarded as insufficient because these studies were not designed as RCTs. At a meeting of the Japanese Society of Nephrology in 2011, the Ministry of Health, Labour and Welfare (MHLW) Progressive Renal Dysfunction Research Group stated that tonsillectomy combined with steroid pulse therapy was found to be more effective than steroid pulse therapy alone in reducing urinary protein in RCTs. This regimen was suggested as a possible treatment option for IgAN. To establish more substantial evidence, the superiority of tonsillectomy combined with steroid pulse therapy should be further investigated.

The efficacy of tonsillectomy has been reported since the 1980s, but different studies have yielded different results owing to differences in the levels of renal dysfunction, urinary protein, and histopathological damage. In the early 2000 s, a retrospective cohort study with a follow-up period of 11 ± 4 years showed no association between tonsillectomy and the rate of progression to ESRD. On the other hand, another study with a longer follow-up of 16 ± 6 years reported a lower incidence of ESRD in the tonsillectomy group than in the non-tonsillectomy group. Because of flaws in the design of previous studies, it is difficult to definitively conclude the efficacy of tonsillectomy. Meanwhile, previous reports show that the tonsillectomy may improve urinary findings in patients with IgAN and may lower the progression of renal dysfunction for a long period of ≥15 years, especially at a relatively early stage, with no serological renal dysfunction or sclerotic lesions in the glomeruli. Previous studies may have shown the efficacy of tonsillectomy in cases of IgAN for a long period, but none of these studies was a RCT. Therefore, according to current clinical practices in Japan, the Guidelines Advisory Committee has decided on a recommendation grade of C1.

RCTs for the effectiveness of cyclophosphamide, azathioprine, cyclosporine, mycophenolate mofetil, and mizoribine for treatment of IgAN have mostly been small-scale studies with a small number of patients. Therefore, it has been difficult to reach a consensus about their effectiveness. Some studies have reported effectiveness in reducing urinary protein level and improving the prognosis of renal function. Further investigations are necessary.

### 3. CQs about immunosuppressive therapy (children)



Children with IgAN can be categorized into 2 broad groups according to clinical or histological severity. For children with mild urinary protein excretion (morning urinary protein/creatinine ratio <1.0), focal mesangial proliferation, and <30 % crescentic glomeruli (mild cases), non-immunosuppressive therapy with RAS blockers and/or Sairei-to (herbal medicine) is recommended. However, for children with severe urinary protein excretion (morning urinary protein/creatinine ratio ≥1.0), moderate or greater mesangial proliferation, crescent formation, adhesions, or sclerotic lesions (any of the above) involving ≥80 % of all the glomeruli or ≥30 % of crescentic glomeruli (severe cases), combination therapy with corticosteroids and non-steroidal immunosuppressive agents, anticoagulants, and antiplatelet agents is highly effective.

In severe childhood IgAN with diffuse mesangial proliferation, combination “cocktail” therapy for 2 years with four drugs, consisting of corticosteroids, non-steroidal immunosuppressive agents (azathioprine), anticoagulants, and antiplatelet agents, is more effective than therapy with corticosteroids alone in reducing proteinuria and preventing the progression of glomerular sclerosis. In addition, combination therapy using azathioprine is associated with a significantly higher 10-year renal survival rate than combined treatment with an anticoagulant and antiplatelet agents. Combination therapy using mizoribine has similar therapeutic effectiveness as combination therapy with azathioprine.

### 4. CQs about supportive therapy (adults)



To evaluate the effectiveness of RAS blockers for treating IgAN, patients with IgAN with urinary protein level ≥1 g/day and CKD stage G1-3b have been enrolled in RCTs. Many studies have reported anti-proteinuric effects of RAS blockers, and 2 studies with a mean follow-up period of ≥5 years reported an improvement in the prognosis of renal function. Therefore, RAS blockers are recommended for patients with IgAN with urinary protein level ≥1 g/day and CKD stage 1–3b. The effectiveness of RAS blockers in patients with IgAN with urinary protein level <1 g/day has not been fully evaluated. Combination therapy with ACE inhibitors, angiotensin receptor blockers (ARBs), aldosterone antagonists, and renin inhibitors is an issue requiring further investigation. RAS blockers are contraindicated in women who are pregnant or trying to conceive. RAS blockers for IgAN patients without hypertension are off-label use in Japan.

Only few studies have evaluated the effectiveness of antiplatelet agents (dipyridamole, dilazep, ticlopidine, and aspirin) and anticoagulants (warfarin) in adult IgAN. Therefore, their effectiveness is currently unknown. In a multicenter double-blind RCT conducted in Japan by Tojo et al. in the 1980s, a subgroup analysis showed that dipyridamole and dilazep was effective in reducing proteinuria in patients with IgAN. The effectiveness of dipyridamole and dilazep in patients with IgAN should be further investigated in meticulously planned RCTs.

Only 6 RCTs have evaluated the effectiveness of n-3 fatty acids (fish oil) in IgAN, so it has been difficult to reach a consensus about their effectiveness. In the largest and longest study involving 106 patients with IgAN, fish oil was reported to inhibit the progression to ESRD. However, other small-scale short-term studies have not confirmed the effectiveness of fish oil. Further investigation is necessary.

### 5. CQs about lifestyle and dietary guidance in IgA nephropathy



There is no direct evidence showing that salt intake restriction is effective in patients with IgAN. However, in an intervention study of non-diabetic patients with CKD, limited salt intake helped reduce blood pressure and decrease urinary protein excretion. Because blood pressure and urinary protein are related to prognosis in IgAN, limiting salt intake is probably beneficial in these patients. Furthermore, in a cohort study involving non-diabetic patients with CKD, higher salt intake increased the risk of renal dysfunction and accelerated progression to ESRD, suggesting that limited salt intake is also a treatment option in patients with IgAN. Nevertheless, these studies were conducted outside Japan and included patients without IgAN. Therefore, the effectiveness and indications for salt intake restriction in Japanese patients with IgAN requires further investigation.

No direct evidence exists to show that protein restriction is effective in patients with IgAN. However, in a meta-analysis involving patients with CKD, limited protein intake did reduce the risk of ESRD and death. On the other hand, limited protein intake has not been shown to reduce the rate of decline in the glomerular filtration rate (GFR). Moreover, severe protein restriction may increase the risk of death, especially after dialysis is initiated. Factors such as age and overall condition differ among individual patients with IgAN, so uniform protein restrictions should not be recommended to all patients. The indications for protein restrictions should be decided on the basis of comprehensive assessments, the risk of progressive renal dysfunction, and adherence to treatment. In addition, when recommending protein restrictions, caution should be taken to avoid malnutrition.

Patients with IgAN who are obese have higher levels of proteinuria, exhibit greater histological injury on renal biopsy associated with obesity, and have a higher future risk of hypertension and progressive renal dysfunction than non-obese patients with IgAN. Moreover, obesity increases the risk of the development and exacerbation of lifestyle-related diseases such as hypertension, diabetes, and lipid disorders. These lifestyle-related diseases adversely affect the prognosis in patients with kidney disease. Therefore, weight loss should be recommended to obese patients with IgAN. However, there is currently no evidence suggesting that weight reduction helps control renal dysfunction or reduce the level of urinary protein. Further investigation is needed.

Exercise is reported to transiently increase urinary protein excretion in patients with IgAN, On the other hand, exercise therapy improves maximum oxygen uptake in patients with CKD. Although there is insufficient evidence regarding the effects and indications for exercise therapy in CKD, exercise restriction should not be recommended to patients with IgAN. Meanwhile, with regard to strenuous exercise, almost no evidence exists regarding the association of exercise in CKD, with a relatively rapid decline in GFR or in CKD with severe nephrotic-level proteinuria. Therefore, the indications for exercise therapy or exercise restrictions should be based on comprehensive assessment of each individual patient’s condition. These patients will require careful follow-up.

In a cohort study of patients with IgAN in Japan and overseas, smoking and the number of cigarettes smoked per day at the time of renal biopsy were associated with a decrease in renal function. In a cohort study of the general population in Japan and overseas, current smoking was related to decreased renal function and renal failure, positive findings for proteinuria, and albuminuria. Therefore, patients with IgAN should be advised to stop smoking to prevent decreased renal function and increased proteinuria. Previous smoking is also a risk factor for renal failure and albuminuria, but the lower risks are associated with previous smoking than current smoking; therefore, smoking cessation in current smokers may potentially prevent any further decline in renal function or increase in proteinuria.

In addition, the number of cigarettes per day and cumulative smoking (pack/years) are risk factors for decreased renal function. Therefore, even when patients are unable to quit smoking, reducing the number of cigarettes smoked per day may help reduce the risk of renal dysfunction. Although no direct evidence exists to show that smoking cessation or reduction prevents deterioration in renal function or exacerbation of proteinuria, smoking itself is an important risk factor not only for renal prognosis but also for lung cancer, COPD, and cardiovascular disease. Therefore, it is important for healthcare providers to provide guidance for smoking cessation.

### 6. Adverse events associated with steroid therapy and immunosuppressive agents

To date, no study has shown a high rate of serious adverse events associated with steroid therapy in adult patients with IgAN. However, because it is not certain whether these findings were based on sufficient disclosure of information, an assessment of risk factors for adverse events and preventive measures should be conducted before starting steroid therapy. Meanwhile, the indications for immunosuppressive therapy must be carefully decided after weighing the potential benefits against the potential risks, because immunosuppressive agents induce serious adverse events in some cases. A “cocktail” therapy involving a combination of corticosteroids and immunosuppressive agents has been shown to be effective in children with IgAN. However, the safety of this combination therapy must be further confirmed, because for some patients, this therapy was discontinued owing to the adverse events. Tonsillectomy in patients with IgAN has a very low rate of serious complications. Cooperation between otolaryngologists and nephrologists is essential for prevention of complications during surgery for patients on immunosuppressive therapy after kidney transplantation and to detect any remnant tonsillar tissue in the patients.

#### **Acknowledgments**

All authors are advisory committee members of Clinical Guidelines for IgA Nephropathy 2014. Committee chairman: Yukio Yuzawa. Committee members: Maki Urushihara, Shoji Kagami, Ritsuko Katafuchi, Hiroshi Kitamura, Hiroyuki Komatsu, Masashi Goto, Shuji Kondo, Mitsuhiro Sato, Kazuo Takahashi, Miki Takahara, Makoto Tomita , Yasuaki Harabuch, Yoshihide Fujigaki, Takashi Yasuda, Yoshinari Yasuda, Ryohei Yamamoto. Chief Chairman of the Clinical Practice Guidelines for Progressive Kidney Diseases: Kenjiro Kimura. Leader of the Research for Progressive Kidney Diseases of the Ministry of Health, Labour and Welfare: Seiichi Matsuo. Cooperative Medical Society: The Japanese Society for Pediatric Nephrology, The Oto-Rhino-Laryngological Society of Japan.

**Bibliography**

I. Introduction

1. Definition and background

1. Berger J, et al. J Urol Nephro(Paris)1968;74:694–5.
2. Baehr G. JAMA 1926;86:1001–4.
3. Bates RC, et al. Am J Med 1957;23:510–28.
4. Galle P, et al. J Urol Nephro(Paris)1962;68:123–7.
5. Cattran DC, et al. Working Group of the International IgA Nephropathy Network and the Renal Pathology Society. Kidney Int 2009;76:534–45.
6. Chauveau D, et al. Contrib Nephlol 1993;104:1–5.
7. Koyama A, et al. Am J Kidney Dis 1997;29:526–32

2. Pathogenesis and pathophysiology

1. Overview

1. Coppo R, et al. J Nephrol 2010;23:626–32.
2. Suzuki Y, et al. Clin Dev Immunol 2011;2011:639074.
3. Donadio JV, et al. N Engl J Med 2002;347:738–48.
4. Boyd JK, et al. Kidney Int 2012;81:833–43.
5. Narita I, et al. Clin Exp Nephrol 2008;12:332–8.
6. Floege J. Am J Kidney Dis 2011;58:992–1004.
7. Suzuki H, et al. J Am Soc Nephrol 2011;22:1795–803.
8. Mestecky J, et al. Annu Rev Pathol 2013;8:217–40.
9. Novak J, et al. Semin Immunopathol 2012;34:365–82.
10. Gharavi AG, et al. Nat Genet 2011;43:321–7.
11. Kiryluk K, et al. PLoS Genet 2012;8:e1002765.
12. Berger J, et al. Kidney Int 1975;7:232–41.
13. Berger J. Am J Kidney Dis 1988;12:371–2.
14. Ponticelli C, et al. Kidney Int 2001;60:1948–54.
15. Floege J. Semin Nephrol 2004;24:287–91.
16. Sanfilippo F, et al. Transplantation 1982;33:370–6.
17. Silva FG, et al. Transplantation 1982;33:241–6.
18. Cuevas X, et al. Transplant Proc 1987;19:2208–9.
19. Iwata Y, et al. Intern Med 2006;45:1291–5.
20. Zickerman AM, et al. Am J Kidney Dis 2000;36:E19.
21. Van Der Helm–Van Mil AH, et al. Br J Haematol 2003;122:915–7.
22. Pouria S, et al. Semin Nephrol 2008;28:27–37.
23. Glassock RJ. Curr Opin Nephrol Hypertens 2011;20:153–60.
24. Mestecky J. Am J Kidney Dis 1988;12:378–83.
25. Conley ME, et al. J Clin Invest 1980;66:1432–6.
26. van der Boog PJ, et al. Kidney Int 2005;67:813–21.
27. Suzuki K, et al. Kidney Int 2003;63:2286–94.
28. Moura IC, et al. Semin Nephrol 2008;28:88–95.
29. Hiki Y. Clin Exp Nephrol 2009;13:415–23.
30. Monteiro RC, et al. Annu Rev Immunol 2003;21:177–204.
31. Berthelot L, et al. J Exp Med 2012;209:793–806.
32. Lai KN. Nat Rev Nephrol 2012;8:275–83.
33. Feehally J, et al. J Am Soc Nephrol 2010;21:1791–7.
34. Yu XQ, et al. Nat Genet 2011;44:178–82.
35. Macpherson AJ, et al. Trends Immunol 2012;33:160–7.

2. Genetics

1. Johnston PA, et al. Q J Med 1992;84:619–27.
2. Rambausek M, et al. Pediatr Nephrol 1987;1:416–8.
3. Paterson AD, et al. J Am Soc Nephrol 2007;18:2408–15.
4. Gharavi AG, et al. Nat Genet 2011;43:321–7.
5. Yu XQ, et al. Nat Genet 2011;44:178–82.
6. Kiryluk K, et al. PLoS Genet 2012;8:e1002765.
7. Gharavi AG, et al. Nat Genet 2000;26:354–7.
8. Bisceglia L, et al. Am J Hum Genet 2006;79:1130–4.
9. Karnib HH, et al. Nephrol Dial Transplant 2007;22:772–7.
10. Frascá GM, et al. J Nephrol 2004;17:778–85.
11. Takei T, et al. Am J Hum Genet 2002;70:781–6.
12. Akiyama F, et al. J Hum Genet 2002;47:532–8.
13. Obara W, et al. J Hum Genet 2003;48:293–9.
14. Ohtsubo S, et al. J Hum Genet 2005;50:30–5.
15. Feehally J, et al. J Am Soc Nephrol 2010;21:1791–7.
16. Imielinski M, et al. Nat Genet 2009;41:1335–40.
17. Kiryluk K, et al. Annu Rev Med 2013;64:339–56.
18. Kiryluk K, et al. Pediatr Nephrol 2010;25:2257–68.
19. Gharavi AG, et al. J Am Soc Nephrol 2008;19:1008–14.
20. Tam KY, et al. Kidney Int 2009;75:1330–9.
21. Kiryluk K, et al. Kidney Int 2011;80:79–87.
22. Li GS, et al. Kidney Int 2007;71:448–53.
23. Pirulli D, et al. J Nephrol 2009;22:152–9.
24. Zhu L, et al. Kidney Int 2009;76:190–8.
25. Li GS, et al. Hum Mutat 2007;28:950–7.
26. Malycha F, et al. Nephrol Dial Transplant 2009;24:321–4.
27. Suzuki K, et al. Kidney Int 2003;63:2286–94.
28. Floege J. Am J Kidney Dis 2011;58:992–1004.
29. Miyazaki M. Jpn J Med 1990;29:469–77.

3. Abnormal IgA molecules

1. van der Boog PJ, et al. Kidney Int 2005;67:813–21.
2. Mestecky J, et al. Contrib Nephrol 1993;104:172–82.
3. Allen AC. Nephrol Dial Transplant 1995;10:1121–4.
4. Hiki Y, et al. Contrib Nephrol 1995;111:73–84.
5. Hiki Y, et al. Kidney Int 2001;59:1077–85.
6. Allen AC, et al. Kidney Int 2001;60:969–73.
7. Mattu TS, et al. J Biol Chem 1998;273:2260–72.
8. Mestecky J, et al. Mucosal Immunology, 3rd ed. 2005, pp153–81.
9. Peppard JV, et al. Mucosal Immunology, 3rd ed. 2005, pp195–210.
10. Tarelli E, et al. Carbohydr Res 2004;339:2329–35.
11. Renfrow MB, et al. J Biol Chem 2005;280:19136–45.
12. Iwasaki H, et al. J Biol Chem 2003;278:5613–21.
13. Takahashi K, et al. Mol Cell Proteomics 2010;9:2545–57.
14. Takahashi K, et al. J Proteome Res 2012;11:692–702.
15. Novak J, et al. Semin Nephrol 2008;28:78–87.
16. Ju T, et al. Nature 2005;437:1252.
17. Schachter H, et al. J Biol Chem 1971;246:5321–8.
18. Moldoveanu Z, et al. Kidney Int 2007;71:1148–54.
19. Shimozato S, et al. Nephrol Dial Transplant 2008;23:1931–9.
20. Wada Y, et al. J Proteome Res 2010;9:1367–73.
21. Smith AC, et al. J Am Soc Nephrol 2006;17:1192–9.
22. Suzuki H, et al. J Clin Invest 2008;118:629–39.
23. Malycha F, et al. Nephrol Dial Transplant 2009;24:321–4.
24. Serino G, et al. J Am Soc Nephrol 2012;23:814–24.
25. Yamada K, et al. Nephrol Dial Transplant 2010;25:3890–7.
26. Buck KS, et al. Kidney Int 2008;73:1128–36.
27. Lai KN, et al. Kidney Int 1988;33:584–9.
28. Chui SH, et al. J Clin Immunol 1991;11:219–23.
29. Leung JC, et al. J Lab Clin Med 1999;133:152–60.
30. Amore A, et al. J Am Soc Nephrol 2001;12:1862–71.
31. Leung JC, et al. J Clin Lab Anal 2002;16:11–9.
32. Odani H, et al. Biochem Biophys Res Commun 2000;271:268–74.
33. Xu LX, et al. Kidney Int 2005;68:167–72.
34. Ding JX, et al. Clin Immunol 2007;125:268–74.
35. Maenuma K, et al. J Proteome Res 2009;8:3617–24.
36. Leung JC, et al. Kidney Int 2001;59:277–85.
37. Horie A, et al. Am J Kidney Dis 2003;42:486–96.
38. Hiki Y. Clin Exp Nephrol 2009;13:415–23.

4. Mucosal immunity

1. Feehally J, et al. Kidney Int 1986;30:924–31.
2. Hotta O, et al. Am J Kidney Dis 2001;38:736–43.
3. Xie Y, et al. Kidney Int 2003;63:1861–7.
4. Coppo R, et al. J Nephrol 2010;23:626–32.
5. Boyd JK, et al. Kidney Int 2012;81:833–43.
6. Leinikki PO, et al. Clin Exp Immunol 1987;68:33–8.
7. van den Wall Bake AW, et al. J Clin Invest 1989;84:1070–5.
8. Layward L, et al. Clin Immunol Immunopathol 1993;69:306–13.
9. Barratt J, et al. Am J Kidney Dis 1999;33:1049–57.
10. de Fijter JW, et al. Kidney Int 1996;50:952–61.
11. Roodnat JI, et al. Nephrol Dial Transplant 1999;14:353–9.
12. van den Wall Bake AW, et al. Clin Exp Immunol 1988;72:321–5.
13. van den Wall Bake AW, et al. Kidney Int 1989;35:1400–4.
14. Harper SJ, et al. J Clin Pathol 1996;49:38–42.
15. Westberg NG, et al. Clin Immunol Immunopathol 1983;26:442–5.
16. Harper SJ, et al. Kidney Int 1994;45:836–44.
17. Kunkel EJ, et al. Nat Rev Immunol 2003;3:822–9.
18. Smith AC, et al. J Am Soc Nephrol 2006;17:3520–8.
19. Barratt J, et al. Nephrol Dial Transplant 2009;24:3620–3.
20. Batra A, et al. Nephrol Dial Transplant 2007;22:2540–8.
21. Buren M, et al. Contrib Nephrol 2007;157:50–5.
22. Gesualdo L, et al. J Immunol 1990;145:3684–91.
23. Zadrazil J, et al. J Nephrol 2006;19:382–6.
24. Chan SM, et al. Br J Dermatol 2007;156:143–7.
25. Trimarchi HM, et al. Am J Nephrol 2001;21:400–5.
26. Forshaw MJ, et al. Int J Colorectal Dis 2005;20:463–5.
27. de Moura CG, et al. J Clin Rheumatol 2006;12:106–7.
28. Wang J, et al. J Clin Invest 2004;113:826–35.
29. Coppo R, et al. Kidney Int 2009;75:536–41.
30. Coppo R, et al. Clin Exp Immunol 2010;159:73–81.
31. Qin W, et al. Nephrol Dial Transplant 2008;23:1608–14.
32. Suzuki H, et al. J Am Soc Nephrol 2008;19:2384–95.
33. Sato D, et al. Nephrol Dial Transplant 2012;27:1090–7.
34. Fujihashi K, et al. J Exp Med 1996;183:1929–35.
35. Toyabe S, et al. Clin Exp Immunol 2001;124:110–7.
36. Olive C, et al. Kidney Int 1997;52:1047–53.
37. Wu H, et al. Kidney Int 1999;55:109–19.
38. Buck KS, et al. Clin Exp Immunol 2002;127:527–32.
39. Cox SN, et al. Kidney Int 2012;82:548–60.
40. Goto T, et al. Clin Immunol 2008;126:260–9.
41. McCarthy DD, et al. J Clin Invest 2011;121:3991–4002.
42. Macpherson AJ, et al. Trends Immunol 2012;33:160–7.
43. Jin J, et al. Nat Immunol 2012;13:1101–9.
44. Marquina R, et al. J Immunol 2004;172:7177–85.
45. Gonzalez J, et al. J Immunol 2007;178:2778–86.
46. Murakata H, et al. Acta Otolaryngol 1999;119:384–91.
47. Suzuki S, et al. Lancet 1994;343:12–6.
48. Fujieda S, et al. Clin Immunol 2000;95:235–43.
49. Nagy J, et al. Scand J Immunol 1988;27:393–9.
50. Bene MC, et al. Nephron 1991;58:425–8.
51. Egido J, et al. Clin Exp Immunol 1984;57:101–6.
52. Inoue T, et al. Clin Immunol 2010;136:447–55.
53. Horie A, et al. Am J Kidney Dis 2003;42:486–96.
54. Xie Y, et al. Kidney Int 2004;65:1135–44.
55. Baumgarth N. Nat Rev Immunol 2011;11:34–46.
56. Kodama S, et al. Clin Exp Immunol 2001;123:301–8.
57. Yuling H, et al. J Am Soc Nephrol 2008;19:2130–9.
58. Nozawa H, et al. Clin Exp Immunol 2008;151:25–33.
59. Johnson RJ, et al. Am J Kidney Dis 2003;42:575–81.
60. Emancipator SN, et al. J Exp Med 1983;157:572–82.
61. Ichinose H, et al. Clin Exp Immunol 1996;103:125–32.
62. Ebihara I, et al. Nephrol Dial Transplant 2001;16:1783–9.
63. Yano N, et al. J Clin Immunol 1997;17:396–403.
64. Lim CS, et al. Nephrol Dial Transplant 2001;16:269–75.
65. Suzuki H, et al. Kidney Int 2007;72:319–27.
66. Suzuki Y, et al. Contrib Nephrol 2007;157:70–9.
67. Mora JR, et al. Mucosal Immunol 2008;1:96–109.
68. Yamada K, et al. Nephrol Dial Transplant 2010;25:3890–7.
69. Coppo R, et al. Clin Nephrol 1990;33:72–86.
70. Waldo FB, et al. Lancet 1989;1:129–31.
71. Iwama H, et al. Am J Kidney Dis 1998;32:785–93.
72. Koyama A, et al. Kidney Int 1995;47:207–16.
73. Koyama A, et al. Kidney Int 2004;66:121–32.
74. Schmitt R, et al. Am J Pathol 2010;176:608–18.
75. Fornasieri A, et al. Br Med J(Clin Res Ed)1987;295:78–80.
76. Collin P, et al. Am J Gastroenterol 2002;97:2572–6.
77. Pierucci A, et al. Am J Kidney Dis 2002;39:1176–82.
78. Sategna–Guidetti C, et al. Gut 1992;33:476–8.
79. Jackson S, et al. Clin Exp Immunol 1992;89:315–20.
80. Russell MW, et al. J Clin Immunol 1986;6:74–86.
81. Feehally J, et al. Pediatr Nephrol 1987;1:581–6.
82. Sato M, et al. Clin Exp Immunol 1988;73:295–9.
83. Smerud HK, et al. Nephrol Dial Transplant 2009;24:2476–81.
84. Ferri C, et al. Nephrol Dial Transplant 1993;8:1193–8.

5. IgA1 glomerular deposition

1. Tomino Y, et al. Am J Kidney Dis 1982;1:276–80.
2. Monteiro RC, et al. Kidney Int 1985;28:666–71.
3. Conley ME, et al. J Clin Invest 1980;66:1432–6.
4. van der Boog PJ, et al. Kidney Int 2005;67:813–21.
5. Tomino Y, et al. Clin Exp Immunol 1982;49:419–25.
6. Hiki Y, et al. Kidney Int 2001;59:1077–85.
7. Allen AC, et al. Kidney Int 2001;60:969–73.
8. Kokubo T, et al. J Am Soc Nephrol 1997;8:915–9.
9. Tomana M, et al. Kidney Int 1997;52:509–16.
10. Tomana M, et al. J Clin Invest 1999;104:73–81.
11. Suzuki H, et al. J Clin Invest 2009;119:1668–77.
12. Berthoux F, et al. J Am Soc Nephrol 2012;23:1579–87.
13. Novak J, et al. Semin Nephrol 2008;28:78–87.
14. Suzuki H, et al. J Am Soc Nephrol 2011;22:1795–803.
15. Levinsky RJ, et al. Lancet 1979;2:1100–3.
16. Hall RP, et al. Clin Exp Immunol 1980;40:431–7.
17. Hall RP, et al. Clin Exp Immunol 1981;45:234–9.
18. Tissandie E, et al. Kidney Int 2011;80:1352–63.
19. Novak J, et al. Kidney Int 2005;67:504–13.
20. Glassock RJ. Curr Opin Nephrol Hypertens 2011;20:153–60.
21. Mauer SM, et al. J Exp Med 1973;137:553–70.
22. Couser WG. J Am Soc Nephrol 2012;23:381–99.
23. Lai KN. Nat Rev Nephrol 2012;8:275–83.
24. Hiki Y. Clin Exp Nephrol 2009;13:415–23.
25. Monteiro RC, et al. Annu Rev Immunol 2003;21:177–204.
26. Moura IC, et al. J Exp Med 2001;194:417–25.
27. Moura IC, et al. J Am Soc Nephrol 2004;15:622–34.
28. McDonald KJ, et al. Biochem Biophys Res Commun 2002;290:438–42.
29. Diven SC, et al. Kidney Int 1998;54:837–47.
30. Leung JC, et al. J Am Soc Nephrol 2000;11:241–9.
31. Novak J, et al. Kidney Int 2002;62:465–75.
32. Westerhuis R, et al. J Am Soc Nephrol 1999;10:770–8.
33. Barratt J, et al. Kidney Int 2000;57:1936–48.
34. Kaneko Y, et al. Int Immunol 2012;24:219–32.
35. Launay P, et al. J Exp Med 2000;191:1999–2009.
36. Pleass RJ, et al. J Biol Chem 1999;274:23508–14.
37. van der Boog PJ, et al. Nephrol Dial Transplant 2004;19:2729–36.
38. van der Boog PJ, et al. Kidney Int 2003;63:514–21.
39. Moura IC, et al. J Am Soc Nephrol 2005;16:2667–76.
40. Haddad E, et al. J Am Soc Nephrol 2003;14:327–37.
41. Berthelot L, et al. J Exp Med 2012;209:793–806.
42. Kawabata H, et al. J Biol Chem 1999;274:20826–32.
43. Itoh M, et al. J Histochem Cytochem 2011;59:180–7.
44. Coppo R, et al. Contrib Nephrol 1993;104:162–71.
45. Kokubo T, et al. J Am Soc Nephrol 1998;9:2048–54.
46. Sano T, et al. Nephrol Dial Transplant 2002;17:50–6.
47. Leung JC, et al. Kidney Int 2001;59:277–85.
48. Leung JC, et al. J Clin Lab Anal 2002;16:11–9.
49. Nishie T, et al. Am J Pathol 2007;170:447–56.
50. Jennette JC, et al. Am J Kidney Dis 1991;18:466–71.
51. Zheng F, et al. Nat Med 1999;5:1018–25.
52. Narita I, et al. Kidney Int 2002;61:1853–8.
53. Yong D, et al. Am J Kidney Dis 2006;48:1–7.
54. Coppo R, et al. Am J Kidney Dis 2002;40:495–503.
55. Hiki Y, et al. J Am Soc Nephrol 1996;7:955–60.
56. Hiki Y, et al. J Am Soc Nephrol 1999;10:760–9.
57. Iwase H, et al. Biochem Biophys Res Commun 1999;261:472–7.
58. Mole CM, et al. Nephron 1995;71:75–8.
59. Oortwijn BD, et al. Kidney Int 2006;69:1131–8.
60. Zhang JJ, et al. Nephrol Dial Transplant 2008;23:207–12.
61. Delacroix DL, et al. J Clin Invest 1983;71:358–67.
62. van den Wall Bake AW, et al. Am J Kidney Dis 1988;12:410–4.
63. Harper SJ, et al. Am J Kidney Dis 1994;24:888–92.
64. van den Wall Bake AW, et al. Clin Exp Immunol 1988;72:321–5.
65. van den Wall Bake AW, et al. Kidney Int 1989;35:1400–4.
66. Pouria S, et al. Semin Nephrol 2008;28:27–37.
67. Roccatello D, et al. Am J Kidney Dis 1989;14:354–60.
68. Stockert RJ. Physiol Rev 1995;75:591–609.
69. Leung JC, et al. J Lab Clin Med 1999;133:152–60.
70. Grossetete B, et al. Kidney Int 1998;53:1321–35.
71. van Zandbergen G, et al. Nephrol Dial Transplant 1998;13:3058–64.
72. Montenegro V, et al. J Rheumatol 2000;27:411–7.
73. Grossetete B, et al. AIDS 1995;9:229–34.
74. Silvain C, et al. J Immunol 1995;155:1606–18.
75. Hotta O, et al. Am J Kidney Dis 2002;39:493–502.
76. Imasawa T, et al. Kidney Int 1999;56:1809–17.
77. Gomez–Guerrero C, et al. J Immunol 1994;153:5247–55.
78. Barratt J, et al. J Am Soc Nephrol 2005;16:2088–97.
79. Lamm ME, et al. Am J Pathol 2008;172:31–6.

6. Glomerular damage

1. Barratt J, et al. Semin Nephrol 2011;31:349–60.
2. Lai KN. Nat Rev Nephrol 2012;8:275–83.
3. Novak J, et al. Kidney Int 2002;62:465–75.
4. Lopez–Armada MJ, et al. J Immunol 1996;157:2136–42.
5. Novak J, et al. Kidney Int 2005;67:504–13.
6. van den Dobbelsteen ME, et al. Kidney Int 1994;46:512–9.
7. Moura IC, et al. J Am Soc Nephrol 2005;16:2667–76.
8. Oortwijn BD, et al. J Am Soc Nephrol 2006;17:3529–39.
9. Lai KN, et al. J Am Soc Nephrol 2003;14:3127–37.
10. Leung JC, et al. Nephrol Dial Transplant 2003;18:36–45.
11. Coppo R, et al. Kidney Int 2010;77:417–27.
12. Amore A, et al. J Am Soc Nephrol 2001;12:1862–71.
13. Amore A, et al. Am J Kidney Dis 2000;36:1242–52.
14. Lai KN, et al. Kidney Int 2004;66:1403–16.
15. Miyake–Ogawa C, et al. Am J Nephrol 2005;25:1–12.
16. Tamouza H, et al. Kidney Int 2012;82:1284–96.
17. Kim MJ, et al. J Immunol 2012;189:3751–8.
18. Rauterberg EW, et al. Kidney Int 1987;31:820–9.
19. Stad RK, et al. Clin Exp Immunol 1993;92:514–21.
20. Matsuda M, et al. Nephron 1998;80:408–13.
21. Roos A, et al. J Immunol 2001;167:2861–8.
22. Roos A, et al. J Am Soc Nephrol 2006;17:1724–34.
23. Zwirner J, et al. Kidney Int 1997;51:1257–64.
24. Kim SJ, et al. PLoS One 2012;7:e40495.
25. Montinaro V, et al. J Am Soc Nephrol 1997;8:415–25.
26. Abe K, et al. Nephron 2001;87:231–9.
27. Abe K, et al. Kidney Int 1998;54:120–30.
28. Endo M, et al. Nephrol Dial Transplant 1998;13:1984–90.
29. Lhotta K, et al. Nephrol Dial Transplant 1999;14:881–6.
30. Floege J. Nephron 2002;91:582–7.
31. Asao R, et al. Clin J Am Soc Nephrol 2012;
32. Remuzzi G, et al. N Engl J Med 1998;339:1448–56.
33. Lai KN, et al. Nephrol Dial Transplant 2009;24:62–72.
34. El Karoui K, et al. Kidney Int 2011;79:643–54.
35. Hill GS, et al. Kidney Int 2011;79:635–42.
36. Cook HT. Kidney Int 2011;79:581–3.
37. Tam KY, et al. Am J Physiol Renal Physiol 2010;299:F359–68.
38. Chan LY, et al. Kidney Int 2005;67:602–12.
39. Xiao J, et al. Clin Immunol 2009;132:266–76.
40. Wang L, et al. J Lab Clin Med 2003;142:313–21.
41. Chan LY, et al. J Am Soc Nephrol 2005;16:2306–17.

II. Diagnosis

1. Diagnosis

1. Nakayama K, et al. J Clin Lab Anal 2008;22:114–8.
2. Dische FE, et al. Am J Nephrol 1985;5:103–9.
3. Aarons I, et al. Clin Nephrol 1989;32:151–8.
4. Packham DK. Nephrology(Carlton)2007;12:481–6.
5. Donadio JV, et al. N Engl J Med 2002;347:738–48.
6. Saulsbury FT. Medicine(Baltimore)1999;78:395–409.

2. Clinical manifestations and laboratory findings

1. Ponticelli C, et al. Kidney Int 2001;60:1948–54.
2. Sakai H, et al. The Achievements of Progressive Renal Diseases Research, Research on intractable disease, the Ministry of Health, Labour and Welfare of Japan 1995. 1996;1–5 (Japanese).
3. Floege J, et al. J Am Soc Nephrol 2000;11:2395–403.
4. Donadio JV, et al. N Engl J Med 2002;347:738–48.
5. Coppo R, et al. J Nephrol 2005;18:503–12.
6. D’Amico G. Am J Kidney Dis 1988;12:353–7.
7. D’Amico G. Nephron 1985;41:1–13.
8. D’Amico G, et al. Medicine (Baltimore)1985;64:49–60.
9. D’Amico G. Q J Med 1987;6:709–27.
10. Neelakantappa K, et al. Kidney Int 1988;33:716–21.
11. Perez–Fontan M, et al. Am J Nephrol 1986;6:482–6.
12. Research Group on Progressive Chronic Renal Disease. Nephron 1999;82:205–13.
13. Sugiyama H, et al. Clin Exp Nephrol 2011;15:493–503.
14. Matutani S, et al. Acta Otolaryngol Suppl 2004;555:58–61.
15. Yamabe H, et al. Acta Otolaryngol Suppl 1996;523:169–71.
16. Clinical guides for immunoglobulin A (IgA) nephropathy in Japan, third version. Nihon Jinzo Gakkai Shi. 2011; 53(2):123-35.
17. Cohen RA, et al. N Engl J Med 2003;348:2330–8.
18. Birch DF, et al. Clin Nephrol 1983;20:78–84.
19. Murakami S, et al. J Urol 1990;144:99–101.
20. Grossfeld GD, et al. Am Fam Physician 2001;63:1145–54.
21. Philibert D, et al. Semin Nephrol 2008;28:10–7.
22. Lai KN, et al. Am J Clin Pathol 1986;86:716–23.
23. Sinnassamy P, et al. Am J Kidney Dis 1985;5:267–9.
24. Moriyama T, et al. Int Urol Nephrol 2012;44:1177–84.
25. Kim SM, et al. J Korean Med Sci 2009;24 Suppl:S44–9.
26. KDIGO clinical practice guideline for glomerulonephritis. Chapter 10:Immunogloblin A nephropathy. Kindey Int Suppl 2012;2:209–17.
27. Committee for Diagnostic Guidelines of Hematuria. Nihon Jinzo Gakkai Shi. 2006;48 Suppl:1-34
28. Walshe JJ, et al. Am J Med 1984;77:765–7.
29. MacDonald I, et al. Clin Nephrol 1975;3:129–33.
30. Schena FP, et al. Oxford Textbook of Clinical Nephrology. 3rd ed. 2006;1:469–501.
31. Gutiérrez E, et al. Clin J Am Soc Nephrol 2007;2:51–7.
32. Yoshikawa N, et al. Clin Nephrol 1987;28:217–21.
33. Bennett WM, et al. Kidney Int 1983;23:393–400.
34. Praga M, et al. Kidney Int 1985;28:69–74.
35. Delclaux C, et al. Nephrol Dial Transplant 1993;8:195–9.
36. Matousovic K, et al. Nephrol Dial Transplant 2006;21:2478–84.
37. Obara T, et al. Clin Exp Nephrol 2012;16:713–21.
38. Nakamura T, et al. Am J Nephrol 2005;25:447–50.
39. Moon PG, et al. Proteomics 2011;11:2459–75.
40. Tomino Y, et al. J Clin Lab Anal 2000;14:220–3.
41. Miyazaki R, et al. Clin Nephrol 1984;21:335–40.
42. Wyatt RJ, et al. Kidney Int 1987;31:1019–23.
43. Maeda A, et al. J Clin Lab Anal 2003;17:73–6.
44. Nakayama K, et al. J Clin Lab Anal 2008;22:114–8.
45. Jones CL, et al. Kidney Int 1990;38:323–31.
46. Allen AC, et al. Clin Exp Immunol 1995;100:470–4.
47. Tomana M, et al. 1997;52:509–16.
48. Hiki Y, et al. Kidney Int 2001;59:1077–85.
49. Moldoveanu Z, et al. Kidney Int 2007;71:1148–54.
50. Gharavi AG, et al. J Am Soc Nephrol 2008;19:1008–14.
51. Czerkinsky C, et al. J Clin Invest 1986;77:1931–8.
52. Coppo R, et al. Clin Nephrol 1995;43:1–13.
53. Schena FP, et al. Clin Exp Immunol 1989;77:15–20.
54. Allen AC, et al. Kidney Int 2001;60:969–73.
55. Suzuki H, et al. J Clin Invest 2009;119:1668–77.
56. Hastings MC, et al. Clin J Am Soc Nephrol 2010;5:2069–74.
57. Donadio JV, et al. Nephrol Dial Transplant 2002;17:1197–203.
58. D’Amico G, et al. Q J Med 1986;59:363–78.
59. Reich HN, et al. J Am Soc Nephrol 2007;18:3177–83.
60. Working Group of the International IgA Nephropathy Network and the Renal Pathology Society. Kidney Int 2009;76:534–45.
61. Herzenberg AM, et al. Kidney Int 2011;80:310–7.
62. Hsu SI, et al. Kidney Int 2000;57:1818–35.
63. Karnib HH, et al. Nephrol Dial Transplant 2007;22:772–7.
64. Schena FP, et al. J Am Soc Nephrol 2002;13:453–60.
65. Gharavi AG, et al. Nat Genet 2000;26:354–7.

3. Pathological findings

1. Sugiyama H, et al. Clin Exp Nephrol 2013;17:155–73.
2. Hennigar RA, et al. Silva’s Diagnostic Renal Pathology. Cambridge 2009;pp127–77.
3. Zidar N, et al. Kidney Int 1992;42:1444–9.
4. Working Group of the International IgA Nephropathy Network and the Renal Pathology Society, The Oxford classification of IgA nephropathy:rationale, clinicopathological correlations, and classification. Kidney Int 2009;76:534–45.
5. Working Group of the International IgA Nephropathy Network and the Renal Pathology Society, The Oxford classification of IgA nephropathy:pathology definitions, correlations, and reproducibility. Kidney Int 2009;76:546–56.
6. Clinical guides for immunoglobulin A (IgA) nephropathy in Japan, third version. Nihon Jinzo Gakkai Shi. 2011; 53(2):123-35.
7. Histological atlas of immunoglobulin (IgA) nephropathy. Nihon Jinzo Gakkai Shi. 2011; 53(5): 655-66. (Japanese)
8. Hass M. Heptinstall’s Pathology of the Kidney, 6th ed., vol. 1 (Lippincott Williams & Wilkins) 2007;423–86.
9. Suzuki K, et al. Kidney Int 2003:63:2286–94.
10. D’Amico G. Semin Nephrol 2004;24:179–96.
11. Herzenberg AM, et al. Kidney Int 2011;80:310–7.
12. Shi SF, et al. Clin J Am Soc Nephrol 2011;6:2175–84.
13. Yau T, et al. Am J Nephrol 2011;34:435–44.
14. Kang S, et al. Nephrol Dial Transplant 2012;27:252–8.
15. Alamartine E, et al. Clin J Am Soc Nephrol 2011;6:2384–88.
16. Tsuboi N, et al. Clin J Am Soc Nephrol 2010;5:39–44.
17. Haas M. Am J Kidney Dis 1997;29:829–42.
18. Katafuchi R, et al. Clin J Am Soc Nephrol 2011;6:2806–13.
19. Walsh M, et al. Clin J Am Soc Nephrol 2010;5:423–30.
20. Bellur SS, et al. Nephrol Dial Transplant 2011;26:2533–8.
21. Working group of the international IgA nephropathy network and the Renal Pathology Society, The Oxford IgA nephropathy clinicopathological classification is valid for children as well as adults. Kidney Int 2010;77:921–7.
22. Ikezumi Y, et al. Nephrol Dial Transplant 2006;21:3466–74.
23. Shima Y, et al. Pediatr Nephrol 2012;27:783–92.
24. Halling SE, et al. Nephrol Dial Transplant 2012;27:715–22

4. Classification

1. Lee SM, et al. Hum Pathol 1982;13:314–22.
2. Meadow SR, et al. Q J Med 1972;41:241–58.
3. Lee HS, et al. Clin Nephrol 1987;27:131–40.
4. Lee HS, et al. Nephrol Dial Transplant 2005;20:342–8.
5. Haas M. Am J Kidney Dis 1997;29:829–42.
6. Manno C, et al. Am J Kidney Dis 2007;49:763–75.
7. Alamartine E, et al. Clin Nephrol 1990;34:45–51.
8. Radford MG Jr, et al. J Am Soc Nephrol 1997;8:199–207.
9. Shigematsu H. Pathol Int 1997;47:194–202.
10. Katafuchi R, et al. Clin Nephrol 1998;49:1–8.
11. Magistroni R, et al. J Nephrol 2006;19:32–40.
12. Okonogi H, et al. Nephron Clin Pract 2011;118:c292–300.
13. Wakai K, et al. Nephrol Dial Transplant 2006;21:2800–8.
14. Goto M, et al. Nephrol Dial Transplant 2009;24:3068–74.
15. Goto M, et al. Nephrol Dial Transplant 2009;24:1242–7.
16. Working Group of the International IgA Nephropathy Network and the Renal Pathology Society. Kidney Int 2009;76:546–56.
17. Working Group of the International IgA Nephropathy Network and the Renal Pathology Society. Kidney Int 2009;76:534–45.
18. Working Group of the International IgA Nephropathy Network and the Renal Pathology Society. Kidney Int 2010;77:921–7.
19. Herzenberg AM, et al. Kidney Int 2011;80:310–7.
20. Yau T, et al. Am J Nephrol 2011;34:435–44.
21. Shi SF, et al. Clin J Am Soc Nephrol 2011;6:2175–84.
22. Alamartine E, et al. Clin J Am Soc Nephrol 2011;6:2384–8.
23. Katafuchi R, et al. Clin J Am Soc Nephrol 2011;6:2806–13.
24. Kang SH, et al. Nephrol Dial Transplant 2012;27:252–8.
25. Edström Halling S, et al. Nephrol Dial Transplant 2012;27:715–22.
26. Shima Y, et al. Pediatr Nephrol 2012;27:783–92.
27. Kawamura. T, et al. J Nephrol 2013;26:350–7.

5. Atypical forms of IgA nephropathy

1. Minimal change nephrotic disease (MCD) with mesangial IgA deposits

1. Mustonen J, et al. Clin Nephrol 1983;20:172.6.
2. Lai KN, et al. Am J Clin Pathol 1986;86:716.23.
3. Lai KN, et al. Clin Nephrol 1986;26:174.80.
4. Fukushi K, et al. Jpn J Nephrol 1988;30:253.8.
5. Kim SM, et al. J Korean Med Sci 2009;24 Suppl:S44.9.

2. Acute kidney injury (AKI) associated with macroscopic hematuria

1. Bennett WM, et al. Kidney Int 1983;23:393–400.
2. Praga M, et al. Kidney Int 1985;28:69–74.
3. Delclaux C, et al. 1993;8:195–9.
4. Kveder R, et al. Ther Apher Dial 2009;13:273–7.
5. Gutiérrez E, et al. Clin J Am Soc Nephrol 2007;2:51–7.

3. Crescentic IgA nephropathy

1. Abuelo JG, et al. Medicine(Baltimore)1984;63:396–406.
2. Roccatello D, et al. Nephrol Dial Transplant 1995;10:2054–9.
3. Chambers ME, et al. J Clin Apher 1999;14:185–7.
4. Tumlin JA, et al. Nephrol Dial Transplant 2003;18:1321–9.
5. Bazzi C, et al. Clin J Am Soc Nephrol 2009;4:929–35.
6. Lai KN, et al. Am J Kidney Dis 1987;10:66–70.
7. Welch TR, et al. Am J Dis Child 1988;142:789–93.
8. Harper L, et al. J Nephrol 2000;13:360–6.
9. Nicholls K, et al. Am J Kidney Dis 1985;5:42–6.
10. Coppo R, et al. Int J Artif Organs 1985;Suppl 2:55–8.
11. McIntyre CW, et al. Clin Nephrol 2001;56:193–8.
12. Tang Z, et al. Am J Nephrol 2002;22:480–6.

III. Epidemiology, prognosis, and follow-up

1. Incidence and prevalence

1. Donadio JV, et al. N Engl J Med 2002;347:738–48.
2. Utsunomiya Y, et al. Pediatr Nephrol 2003;18:511–5.
3. McGrogan A, et al. Nephrol Dial Transplant 2011;26:414–30.

2. Natural course

1. Shen P, et al. Nephron Clin Pract 2007;106:c157–61.
2. Chauveau D, et al. Contrib Nephrol 1993;104:1–5.
3. Koyama A, et al. Am J Kidney Dis 1997;29:526–32.
4. D’Amico G. Semin Nephrol 2004;24:179–96.
5. Kusumoto Y, et al. Clin Nephrol 1987;28:118–24.

3. Changes in prognosis with changes in treatment guidelines

1. Komatsu H, et al. Am J Nephrol 2009;30:19–25.
2. Yata N, et al. Pediatr Nephrol 2008;23:905–12.
3. Asaba K, et al. Intern Med 2009;48:883–90.

4. Clinical predictors of progression at the time of initial examination or renal biopsy

1. Wakai K, et al. Nephrol Dial Transplant 2006;21:2800–8.
2. Manno C, et al. Am J Kidney Dis 2007;49:763–75.
3. Goto M, et al. Nephrol Dial Transplant 2009;24:3068–74.
4. Berthoux F, et al. J Am Soc Nephrol 2011;22:752–61.
5. D’Amico G. Semin Nephrol 2004;24:179–96.
6. Eiro M, et al. Nephron 2002;90:432–41.
7. Ieiri N, et al. Clin Exp Nephrol 2012;16:122–9.

5. Clinical predictors of progression during follow-up

1. Kobayashi Y, et al. Nephrology 1997;3:35–40.
2. Bartosik LP, et al. Am J Kidney Dis 2001;38:728–35.
3. Donadio JV, et al. Nephrol Dial Transplant 2002;17:1197–203.
4. Reich HN, et al. J Am Soc Nephrol 2007;18:3177–83.
5. Berthoux F, et al. J Am Soc Nephrol 2011;22:752–61.
6. Le WB, et al. Nephrol Dial Transplant 2012;27:1479–85.
7. Pozzi C, et al. J Am Soc Nephrol 2004;15:157–63.
8. Cheng J, et al. Int J Clin Pract 2009;63:880–8.
9. Lv J, et al. Am J Kidney Dis 2009;53:26–32.
10. Manno C, et al. Nephrol Dial Transplant 2009;24:3694–701.
11. Hwang HS, et al. Nephrology 2010;15:236–41.
12. Payton CD, et al. Nephrol Dial Transplant 1988;3:138–42.

6. Remission of urinary findings and its significance

1. Hotta O, et al. Am J Kidney Dis 2001;38:736–43.
2. Komatsu H, et al. Clin J Am Soc Nephrol 2008;3:1301–7.
3. Miura N, et al. Clin Exp Nephrol 2009;13:460–6.
4. Kawaguchi T, et al. Nephrology 2010;15:116–23.
5. Tatematsu M, et al. Clin Exp Nephrol 2012:1–9.
6. Hwang HS, et al. Nephrology 2010;15:236–41.
7. Pozzi C, et al. Lancet 1999;353:883–7.
8. Hotta O, et al. Am J Kidney Dis 2002;39:493–502.
9. Matsuzaki K, et al. Clin Exp Nephrol 2013;17:827–33.

7. Follow-up

1. Hotta O, et al. Am J Kidney Dis 2001;38:736–43.
2. Szeto C, et al. Am J Med 2001;110:434–37.
3. Shen P, et al. Nephron Clin Pract 2007;106:c157–61.
4. Goto M, et al. Nephrol Dial Transplant 2009;24:3068–74.
5. Goto M, et al. Nephrol Dial Transplant 2009;24:1242–7.
6. Shen P, et al. Neth J Med 2008;66:242–7.

IV. Treatment

1. A summary of management of IgAN in adults, with a focus on prevention of renal dysfunction

2. Clinical questions (CQs) about immunosuppressive therapy (adults)

CQ 1. Are corticosteroids recommended in IgA nephropathy?

1. Lv J, et al. J Am Soc Nephrol 2012;23:1108–16 (Level 1)
2. Zhou YH, et al. PLoS One 2011;6:e18788 (Level 4)
3. Cheng J. Am J Nephrol 2009;30:315–22 (Level 1)
4. Samuels JA, et al. Cochrane Database Syst Rev 2003;4:CD003965 (Level 3)
5. Lv J, et al. Am J Kidney Dis 2009;53:26–32 (Level 2)
6. Manno C, et al. Nephrol Dial Transplant 2009;24:3694–701 (Level 2)
7. Pozzi C, et al. Lancet 1999;353:883–7 (Level 2)
8. Pozzi C, et al. J Am Soc Nephrol 2004;15:157–63 (Level 2)
9. Lai KN, et al. Clin Nephrol 1986;26:174–80 (Level 2)
10. Julian BA, et al. Contrib Nephrol 1993;104:198–206 (Level 2)
11. Katafuchi R, et al. Am J Kidney Dis 2003;41:972–83 (Level 2)
12. Hogg RJ. Clin J Am Soc Nephrol 2006;1:467–74 (Level 2)
13. Koike M, et al. Clin Exp Nephrol 2008;12:250–5 (Level 2)
14. Shoji T, et al. Am J Kidney Dis 2000;35:194–201 (Level 2)

CQ 2. Is tonsillectomy combined with steroid pulse therapy recommended?

1. Hotta O, et al. Am J Kidney Dis 2001;38:736–43 (Level 4)
2. Kawaguchi T, et al. Nephrology(Carlton)2010;15:116–23 (Level 4)
3. Sato M, et al. Nephron Clin Pract 2003;93:c137–45 (Level 4)
4. Komatsu H, et al. Clin J Am Soc Nephrol 2008;3:1301–7 (Level 3)
5. Miura N, et al. Clin Exp Nephrol 2009;13:460–6 (Level 4)
6. Hotta O, et al. Jpn J Nephrol 1993;35:967–73 (Level 4)
7. Hotta O, et al. Acta Otolaryngol Suppl 1996;523:165–8 (Level 4)
8. Kawamura T, et al. Nephrol Dial Transplant 2014;29:1546–53

CQ 3. Is tonsillectomy (alone) recommended?

1. Iino Y, et al. Acta Otolaryngol Suppl 1993;508:29–35 (Level 4)
2. Kosaka M. Nihon Jibiinkoka Gakkai Kaiho 1998;101:916–23 (Level 4)
3. Rasche FM, et al. Clin Nephrol 1999;51:147–52 (Level 4)
4. Chen, Y, et al. Am J Nephrol 2007;27:170–5 (Level 4)
5. Xie, Y, et al. Kidney Int 2003;63:1861–7 (Level 4)
6. Akagi H, et al. Acta Otolaryngol Suppl 2004;555:38–42 (Level 4)
7. Wang, Y, et al. Nephrol Dial Transplant 2011;26:1923–31 (Level 4)
8. Komatsu H, et al. Ren Fail 2012;34:448–53 (Level 4)
9. Maeda I, et al. Nephrol Dial Transplant 2012;27:2806–13 (Level 4)

CQ 4. Are non-steroidal immunosuppressive agents recommended?

1. Walker RG, et al. Clin Nephrol 1990;34:103–7 (Level 2)
2. Ballardie FW, et al. J Am Soc Nephrol 2002;13:142–8 (Level 2)
3. Pozzi C, et al. J Am Soc Nephrol 2010;21:1783–90 (Level 2)
4. Harmankaya O, et al. Int Urol Nephrol 2002;33:167–71 (Level 2)
5. Lai KN, et al. BMJ 1987;295:1165–8 (Level 2)
6. Frisch G, et al. Nephrol Dial Transplant 2005;20:2139–45 (Level 2)
7. Tang S, et al. Kidney Int 2005;68:802–12 (Level 2)
8. Maes BD, et al. Kidney Int 2004;65:1842–9 (Level 2)
9. Xu G, et al. Am J Nephrol 2009;29:362–7 (Level 1)
10. Xie Y, et al. Am J Med Sci 2011;341:367–72 (Level 2)

3. CQs about immunosuppressive therapy (children)

CQ 5. Is immunosuppressive therapy recommended in childhood IgA nephropathy?

1. Yoshikawa N, et al. Pediatr Nephrol 2001;16:446–57.
2. Yoshikawa N, et al. Nihon Jinzo Gakkai Shi. 1997 Jul;39(5):503–6.
3. Yoshikawa N, et al. J Am Soc Nephrol 1999;10:101–9 (Level 2)
4. Yata N, et al. Pediatr Nephrol 2008;23:905–12 (Level 4)
5. Kamei K, et al. Clin J Am Soc Nephrol 2011;6:1301–7 (Level 2)

CQ 6. Is combination “cocktail” therapy recommended in childhood IgA nephropathy?

1. Yoshikawa N, et al. J Am Soc Nephrol 1999;10:101–9 (Level 2)
2. Yoshikawa N, et al. Clin J Am Soc Nephrol 2006;1:511–7 (Level 2)
3. Kamei K, et al. Clin J Am Soc Nephrol 2011;6:1301–7 (Level 2)
4. Yoshikawa N, et al. Pediatr Nephrol 2008;23:757–63 (Level 4)
5. Pozzi C, et al. J Am Soc Nephrol 2010;21:1783–90.

4. CQs about supportive therapy (adults)

CQ 7. Are RAS blockers recommended in IgA nephropathy?

1. Cheng J, et al. Int J Clin Pract 2009;63:880–8 (Level 1)
2. Reid S, et al. Cochrane Database Syst Rev 2011;3:CD003962 (Level 1)
3. Praga M, et al. J Am Soc Nephrol 2003;14:1578–83 (Level 2)
4. Woo KT, et al. Cell Mol Immunol 2007;4:227–32 (Level 2)
5. Ruggenenti P, et al. Am J Kidney Dis 2000;35:1155–65 (Level 2)
6. Woo KT, et al. Kidney Int 2000;58:2485–91 (Level 2)
7. Park HC, et al. Nephrol Dial Transplant 2003;18:1115–21 (Level 2)
8. Li PK, et al. Am J Kidney Dis 2006;47:751–60 (Level 2)
9. Nakamura T, et al. Am J Nephrol 2000;20:373–9 (Level 2)
10. Coppo R, et al. J Am Soc Nephrol 2007;18:1880–8 (Level 2)
11. Horita Y, et al. Hypertens Res 2004;27:963–70 (Level 2)
12. Nakamura T, et al. Am J Hypertens 2007;20:1195–201 (Level 2)

CQ 8. Are antiplatelet agents recommended in IgA nephropathy?

1. Taji Y, et al. Clin Exp Nephrol 2006;10:268–73 (Level 4)
2. Liu XJ, et al. Intern Med 2011;50:2503–10 (Level 1)
3. Chan MK, et al. Am J Kidney Dis 1987;9:417–21 (Level 2)
4. Lee GSL, et al. Nephrology 1997;3:117–21 (Level 2)
5. Camara S, et al. Nephron 1991;58:13–6 (Level 2)
6. Cheng I, et al. Nephrology 1998;4:19–26 (Level 2)

CQ 9. Are n-3 fatty acids (fish oil) recommended in IgA nephropathy?

1. Miller ER, et al. Am J Clin Nutr 2009;89:1937–45 (Level 4)
2. Bennett WM, et al. Clin Nephrol 1989;31:128–31 (Level 2)
3. Pettersson EE, et al. Clin Nephrol 1994;41:183–90 (Level 2)
4. Donadio JV, Jr., et al. J Am Soc Nephrol 1999;10:1772–7 (Level 2)
5. Alexopoulos E, et al. Ren Fail 2004;26:453–9 (Level 2)
6. Ferraro PM, et al. Nephrol Dial Transplant 2009;24:156–60 (Level 2)
7. Liu LL, et al. Clin Nephrol 2012;77:119–25 (Level 1)
8. Hogg RJ, et al. Clin J Am Soc Nephrol 2006;1:467–74 (Level 2)
9. Reid S, et al. Cochrane Database Syst Rev 2011;3:CD003962 (Level 1)
10. Donadio JV, Jr., et al. N Engl J Med 1994;331:1194–9 (Level 2)
11. Donadio JV Jr., et al. J Am Soc Nephrol 2001;12:791–9 (Level 2)

5. CQs about lifestyle and dietary guidance in IgA nephropathy

CQ 10. Should limitation of salt intake be recommended?

1. Vogt L, et al. J Am Soc Nephrol 2008;19:999–1007 (Level 2)
2. Slagman MC, et al. BMJ 2011;343:d4366 (Level 2)
3. Lin J, et al. Clin J Am Soc Nephrol 2010;5:836–43 (Level 4)
4. Vegter S, et al. J Am Soc Nephrol 2012;23:165–73 (Level 4)
5. Stolarz–Skrzypek K, et al. JAMA 2011;305:1777–85 (Level 4)
6. O’Donnell MJ, et al. JAMA 2011;306:2229–38 (Level 4)
7. Cook NR, et al. BMJ 2007;334:885–8 (Level 4)

CQ 11. Should restricted protein intake be recommended?

1. Pedrini MT, et al. Ann Intern Med 1996;124:627–32 (Level 3)
2. Fouque D, et al. Cochrane Database Syst Rev 2009;3:CD001892 (Level 1)
3. Kasiske BL, et al. Am J Kidney Dis 1998;31:954–61 (Level 1)
4. Koya D, et al. Diabetologia 2009;52:2037–45 (Level 2)
5. Cianciaruso B, et al. Am J Kidney Dis 2009;54:1052–61 (Level 2)
6. Menon V, et al. Am J Kidney Dis 2009;53:208–17 (Level 2)

CQ 12. Should weight loss be recommended?

1. Tanaka M, et al. Nephron Clin Pract 2009;112:c71–8 (Level 4)
2. Bonnet F, et al. Am J Kidney Dis 2001;37:720–7 (Level 4)

CQ 13. Should exercise restriction be recommended?

1. Fuiano G, et al. Am J Kidney Dis 2004;44:257–63 (Level 4)
2. Eidemak I, et al. Nephron 1997;75:36–40 (Level 4)
3. Boyce ML, et al. Am J Kidney Dis 1997;30:180–92 (Level 4)
4. Painter PL, et al. Transplantation 2002;74:42–8 (Level 4)
5. Toyama K, et al. J Cardiol 2010;56:142–6 (Level 4)
6. Pechter U, et al. Int J Rehabil Res 2003;26:153–6 (Level 4)

CQ 14. Should smoking cessation be recommended?

1. Yamamoto R, et al. Am J Kidney Dis 2010;56:313–24 (Level 4)
2. Orth SR, et al. Kidney Int 1998;54:926–31 (Level 4)
3. Hallan SI, et al. Kidney Int 2011;80:516–23 (Level 4)
4. Yamagata K, et al. Kidney Int 2007;71:159–66 (Level 4)
5. Ishizaka N, et al. Hypertens Res 2008;31:485–92 (Level 4)

6. Adverse events associated with steroid therapy and immunosuppressive agents

1. Cheng J, et al. Am J Nephrol 2009;30:315–22 (Level 1)
2. Lv J, et al. J Am Soc Nephrol 2012;23:1108–16. (Level 1)
3. Zhou YH, et al. PLoS One 2011;6:e18788.(Level 1)
4. Weinstein RS. Endocrine 2012;41:183–90.
5. Drescher W, et al. Nephrol Dial Transplant 2011;26:2728–31.
6. Fujimoto S, et al. Am J Nephrol 1990;10:231–6. (Level 4)
7. Ballardie FW, et al. J Am Soc Nephrol 2002;13:142–8.(Level 2)
8. Pozzi C, et al. J Am Soc Nephrol 2010;21:1783–90. (Level 2)
9. Stangou M, et al. Clin Exp Nephrol 2011;15:373–80. (Level 2)
10. Maes BD, et al. Kidney Int 2004;65:1842–9. (Level 2)
11. Tang S, et al. Kidney Int 2005;68:802–12. (Level 2)
12. Frisch G, et al. Nephrol Dial Transplant 2005;20:2139–45.(Level 2)
13. Yoshikawa N, et al. Clin J Am Soc Nephrol 2006;1:511–7.(Level 2)
14. Yoshikawa N, et al. J Am Soc Nephrol 1999;10:101–9.(Level 2)
15. Yoshikawa N, et al. Pediatr Nephrol 2008;23:757–63.(Level 4)
16. Salonen A, et al. Laryngoscope 2002;112:94–8.
17. Heiser C, et al. Laryngoscope 2010;120:2119–24.
18. Heiser C, et al. Laryngoscope 2012;122:1265–6.
19. Windfuhr JP, et al. Eur Arch Otorhinolaryngol 2010;267:289–93.
20. Windfuhr JP, et al. Ann Otol Rhinol Laryngol 2003;112:63–70.
21. Lowe D, et al. Lancet 2004;364:697–702.
22. Walker P, et al. Otolaryngol Head Neck Surg 2007;136(4 Suppl):S27–31.
23. Arnoldner C, et al. Wien Klin Wochenschr 2008;120:336–42.
24. Hessén Söderman AC, et al. Laryngoscope 2011;121:2322–6.
25. Tomkinson A, et al. Laryngoscope 2011;121:279–88.
26. Kennoki T, et al. Transplantation 2009;88:935–41.
27. Kurata N, et al. Nihon Jibiinkoka Gakkai Kaiho.2012 Jan;115(1):29–36.
28. Pratt LW. Trans Am Acad Ophthalmol Otolaryngol 1970;74:1146–54.

